# Toxicokinetics of U-47700, tramadol, and their main metabolites in pigs following intravenous administration: is a multiple species allometric scaling approach useful for the extrapolation of toxicokinetic parameters to humans?

**DOI:** 10.1007/s00204-021-03169-y

**Published:** 2021-10-03

**Authors:** Frederike Nordmeier, Iryna Sihinevich, Adrian A. Doerr, Nadja Walle, Matthias W. Laschke, Thorsten Lehr, Michael D. Menger, Peter H. Schmidt, Markus R. Meyer, Nadine Schaefer

**Affiliations:** 1 Institute of Legal Medicine, Saarland University, 66421 Homburg, Germany; 2 Clinical Pharmacy, Saarland University, 66123 Saarbruecken, Germany; 3 Institute for Clinical and Experimental Surgery, Saarland University, 66421 Homburg, Germany; 4 Department of Experimental and Clinical Toxicology, Institute of Experimental and Clinical Pharmacology and Toxicology, Center for Molecular Signaling (PZMS), Saarland University, 66421 Homburg, Germany

**Keywords:** New synthetic opioids, U-47700, Pigs, Population toxicokinetic modeling, Toxicokinetics, LC–MS/MS

## Abstract

New synthetic opioids (NSOs) pose a public health concern since their emergence on the illicit drug market and are gaining increasing importance in forensic toxicology. Like many other new psychoactive substances, NSOs are consumed without any preclinical safety data or any knowledge on toxicokinetic (TK) data. Due to ethical reasons, controlled human TK studies cannot be performed for the assessment of these relevant data. As an alternative animal experimental approach, six pigs per drug received a single intravenous dose of 100 µg/kg body weight (BW) of U-47700 or 1000 µg/kg BW of tramadol to evaluate whether this species is suitable to assess the TK of NSOs. The drugs were determined in serum and whole blood using a fully validated method based on solid-phase extraction and LC–MS/MS. The concentration–time profiles and a population (pop) TK analysis revealed that a three-compartment model best described the TK data of both opioids. Central volumes of distribution were 0.94 L/kg for U-47700 and 1.25 L/kg for tramadol and central (metabolic) clearances were estimated at 1.57 L/h/kg and 1.85 L/h/kg for U-47700 and tramadol, respectively. The final popTK model parameters for pigs were upscaled via allometric scaling techniques. In comparison to published human data, concentration–time profiles for tramadol could successfully be predicted with single species allometric scaling. Furthermore, possible profiles for U-47700 in humans were simulated. The findings of this study indicate that unlike a multiple species scaling approach, pigs in conjunction with TK modeling are a suitable tool for the assessment of TK data of NSOs and the prediction of human TK data.

**Supplementary Information:**

The online version contains supplementary material available at 10.1007/s00204-021-03169-y.

## Introduction

A large and increasing number of new psychoactive substances (NPS) with similar effects as compared to classical drugs of abuse have been released in Europe for about a decade. The most frequently consumed substance classes are synthetic cannabinoids and cathinones, whereas new synthetic opioids (NSOs) remained absent from the illicit drug market for a relatively long time but have increasingly appeared over the last years (EMCDDA 2019).

For a few years now, one of the most popular NSO U-47700 has emerged on the drugs of abuse market (World Health Organization 2016). It was initially sold via the internet as a legal alternative to common opioid drugs, such as heroin or morphine (Mohr et al. 2016; Rohrig et al. 2017). Being cheaper than heroin and having a more potent receptor binding strength to the µ-opioid receptor than morphine, U-47700 has continuously been consumed, although it was scheduled in many European countries and the USA (Koch et al. 2018; Lehmann et al. 2018).

The much higher binding affinities of NSOs result in strong psychoactive and unpredictable toxic effects, even if small doses are consumed. Numerous cases of intoxications have been reported with life-threatening conditions expressed by the classical triad of opioid intoxications, that is to say, respiratory depression, sedation, and miosis (Coopman et al. 2016; Alzghari et al. 2017; Fleming et al. 2017; Rambaran et al. 2017).

The structure of U-47700 is most likely related to tramadol. Unlike tramadol, whose toxicokinetic (TK) properties have extensively been elucidated using different animal models or controlled human studies with different routes of administration (Murthy et al. 2000; Vullo et al. 2014; Evangelista Vaz et al. 2018), preclinical safety data of U-47700 are still lacking except for data from multiple metabolism studies and one animal study in rats (Solimini et al. 2018; Truver et al. 2020). Yet, TK data are important for the interpretation of analytical data, e.g., with respect to time of intake or concentration in real samples. Due to ethical reasons, these data must usually not be elucidated in controlled human studies. Thus, standardized and controlled animal studies remain the only tool for the assessment of TK properties.

TK studies often include repetitive sampling of body fluids or tissues to establish complete concentration–time profiles and, thus, demand large animals like pigs with sufficient blood volume for multiple blood sampling. Furthermore, the anatomical structure and physiological properties (e.g., cardiovascular, urogenital, digestive system) of pigs correlate with those of humans (Puccinelli et al. 2011). Finally, pigs display a comparable cytochrome P450 (CYP) monooxygenase pattern resulting in a similar metabolism (Anzenbacher et al. 1998), as shown for U-47700 in a previous study (Nordmeier et al. 2020a). In addition, pigs have already been proven to be suitable for TK studies of different synthetic cannabinoids and Δ^9^-tetrahydrocannabinol (Schaefer et al. 2015, 2016, 2017, 2018, 2020).

Therefore, the aims of the present study were to (i) elucidate TK data of U-47700, (ii) examine whether domestic pigs can be used for the prediction of human TK of this substance and tramadol and (iii) evaluate a multiple species allometric scaling approach. For this study, a novel liquid chromatography-tandem mass spectrometry (LC–MS/MS) method was developed and fully validated for pig serum and whole blood. The concentration–time profile of U-47700 after intravenous (i.v.) administration to pigs should be set up and compared to that of tramadol. Thereafter, the concentration–time profiles should be modeled to assess whether this model can predict already published tramadol data in humans.

## Materials and methods

### Chemicals and reagents

Chemicals and reagents used in this study are given in the Electronic Supplementary Material (ESM, S1).

### Experimental preparation

The preparation of the used buffer and drug-free pig blood samples as well as the preparation of stock solutions, calibration standards, and quality control samples was performed according to a previously published study (Nordmeier et al. 2021). The preparations are described in detail in the ESM (S1).

### Sample preparation

SPE was carried out according to a previous study (Nordmeier et al. 2021) and is described in detail in the ESM (S1).

### LC–MS/MS conditions

The LC–MS/MS conditions were already described in a previous study (Nordmeier et al. 2021) and are provided in detail in the ESM (S1).

### Method validation

Validation was carried out according to the guidelines of the Society of Toxicological and Forensic Chemistry (GTFCh) and international guidelines (Peters et al. 2007, 2009).

A full validation was performed for pig serum and whole blood, including selectivity, recovery (RE), matrix effects (ME), process efficiency (PE), determination of limits of detection (LODs), linearity, and lower limits of quantification (LLOQs), intra- and interday accuracy and precision tests, processed sample stability, freeze and thaw stability, and carryover effects. RE, ME, and PE were estimated according to Matuszewski et al. (2003). A detailed description is given in the ESM (S1).

### Animals

As already described in previous studies (Schaefer et al. 2016, 2017, 2018; Nordmeier et al. 2020a, 2021), the experiments were carried out according to the German legislation on protection of animals and the National Institute of Health Guide for the Care and Use of Laboratory Animals (Permission no: 32/2018). For this TK study, 12 domestic male pigs (Swabian Hall strain, mean body weight (BW) 42.8 ± 1.9 kg) were used. The pigs had free access to water and daily standard chow. A night prior to the experiment the animals were kept fasting, but still received water ad libitum.

### Surgical procedure

The surgical procedure was performed according to previous studies (Schaefer et al. 2016, 2017, 2018; Nordmeier et al. 2020a, 2021) and is provided in detail in the ESM (S1). In brief, anesthesia of the animals was done with ketamine/xylazine and maintained with isoflurane under the supply of oxygen. Blood sample collection occurred via a triple-lumen central venous catheter in the jugular vein for a spatial separation of blood sampling and substance administration. Triple-lumen catheter contains three separated catheter tails (lumen) summarized in one cannula for the vein entry. Each lumen has its own catheter clamp and its own outlet in the catheter cannula placed in the vein.

### Study design

As already described (Nordmeier 2020a,b, 2021), the TK study included two different groups. Six pigs received a 100 µg/kg BW dose of U-47700 and six other pigs a 1000 µg/kg BW dose of tramadol. For i.v. drug administration, a stock solution of 4 mg/mL U-47700 was prepared by dissolving the solid compound in ethanol. To reach a 100 µg/kg BW dose, appropriate volumes of 1005–1120 µL were used and filled up with 0.9% sodium chloride to obtain a final volume of 10 mL.

As for tramadol, volumes of 906–1134 µL of a purchased tramadol-HCl solution were administered to obtain a 1000 µg/kg BW dose. Prior to administration, those volumes were filled up with 0.9% sodium chloride as well. Administration of the prepared solutions occurred into the jugular vein within 30 s. Blood sampling (about 10 mL each) was carried out before and 1, 2, 5, 10, 15, 30, 45, 60, 90, 120, 180, 240, 300, 360, 420, and 480 min after the administration via the central venous catheter. To obtain serum, an aliquot of each sample (7 mL) was centrifuged at 1250 g for 15 min. All serum and whole blood samples were stored at  – 20 °C until analysis.

### Calculation of half-lives

The calculation of half-lives (*t*_1/2_) was performed for serum and whole blood data using the non-compartmental method (first-order elimination kinetics). The mean of the measured concentrations was plotted on a semi-logarithmic scale lg *c*(A) against the time after dose *t*. For linear regression, the curve was split into three parts (α-, β-, γ-phase) enabling the best coverage of the data points. Linear regression was performed for each part and each part was described by Eq. 1

1 $$lgc\left( A \right) = lgc_{0} \left( A \right) \, {-}{\text{k}}/{2},.{3}0{3} \times t$$

with *k* as elimination rate constant and *c*_0_ (A) as initial concentration of A.

Based on that, *k* was calculated via the slope of the regression curves and t_1/2_ as shown in Eq. 2:

2 $$t_{1/2} = {\text{ln}}\left( {2} \right) / k$$

The calculation was performed using Microsoft Office Excel 2003 (Redmond, WA, USA).

### Population (pop) TK model development for U-47700 and tramadol

PopTK modeling and simulations as well as model evaluations were performed for serum data of the parent compounds using non-linear mixed-effects modeling techniques implemented in NONMEM® (version 7.4.3, ICON Development Solutions, Ellicott City, MD, USA) (Beal et al. 2009). These techniques allow to estimate the typical values of the model parameters for a population and to quantify inter-individual variability (IIV) and residual (unexplained) variability. The first-order conditional estimation with interaction (FOCE-I) method was used in NONMEM®. IIV was modeled using exponential random effects models. Model selection was based on several criteria, including differences in objective function values (dOFV), visual inspection of goodness-of-fit (GOF) plots, and precision of parameter estimates provided by NONMEM®. One nested model was considered superior to another when the OFV was reduced by 3.84 points (chi-square value, p < 0.05, one degree of freedom). For internal model evaluation, a visual predictive check (VPC) was performed based on 1000 simulations using fixed- and random-effects parameters of the final TK models. Median and 90% confidence interval (CI) simulated serum concentrations were calculated, plotted against time, and overlaid with the observed data. Statistical analyses and data visualization were performed in R version 3.6.1 and higher (The R Foundation for Statistical Computing) (R.C. Team 2018) using Rstudio version 1.2.1335 (RStudio, Inc.) and ggplot2 R package (Wickham 2009). PopTK analyses in NONMEM® were performed using Pirana™ version 2.9.5 as a modeling environment.

### Prediction of human tramadol and U-47700 exposure

To predict human exposure, different allometric scaling techniques using single (pig) and multiple species, selected to cover a wide range of BWs (mouse, rat, rhesus macaque, dog, pig, llama, and horse), were evaluated (Huh et al. 2011). Tramadol TK profiles for species other than a pig (Cox et al. 2011; Knych et al. 2013; Kelly et al. 2015; Jamali et al. 2017; Evangelista Vaz et al. 2018) as well as human i.v. pharmacokinetic (PK) studies (Campanero et al. 1999; Quetglas et al. 2007; Yılmaz and Erdem 2015; United States Patent 2018) used for the evaluation of human exposure predictions were obtained during the literature search, digitized, and are listed in Table S5. An appropriate scaling technique for human predictions was selected based on several statistical and graphical criteria (dOFV, GOF plots, and VPCs). The reported dosing regimens, as well as the subjects’ BW were included in the simulation scenarios. If no BW was stated, the population mean BW was used. For human profiles, which had no BW values stated at all, a mean BW of 70 kg was assumed.

For the multiple species allometric scaling approach, simple allometry with a power function correlating model parameters (such as clearance and volume of distribution) with BW (P = aBW^b^) was used, where *P* is the parameter of interest, *a* is the coefficient and *b* is the allometry exponent, respectively (Huh et al. 2011). In an attempt to improve the overall performance of the simple allometric scaling, different correction factors, such as maximum life-span potential (MLP), brain weight (BRW), liver blood flow (LBF), bile flow, and liver weight (LW) on central clearance were tested, given that the relevant values were available in the literature for the selected species.

For the single allometric scaling approach, the final popTK model parameters for pigs were fixed and at first upscaled via allometric scaling techniques, which incorporated BW as an exponential covariate for all model parameters (Eq. 3) (Huh et al. 2011):

3 $${\text{Parameter}}_{{{\text{human}}}} = {\text{ Parameter}}_{{{\text{pig}}}} \times \left( {BW_{{{\text{human}}}} /BW_{{{\text{pig}}}} } \right)^{b}$$

The allometry exponents (b) fixed at values between 0.6 and 1.6 were tested as the most commonly estimated values for small-molecule drugs used for interspecies scaling found in the literature (Huh et al. 2011). BW_pig_ was set to the mean value of 42.83 kg.

The incorporation of LBF into the model as a correction factor on central clearance helped further optimize the description of human data (Table S5) (Campanero et al. 1999; Quetglas et al. 2007; Yılmaz and Erdem 2015; United States Patent 2018). Hence, the human clearance was estimated, as shown in Eq. 4:

4 $${\text{Clearance}}_{{{\text{human}}}} =\, {\text{Clearance}}_{{{\text{pig}}}} \times \left( {BW_{{{\text{human}}}} /BW_{{{\text{pig}}}} } \right)^{b} \times \, \left( {LBF_{{{\text{human}}}} /LBF_{{{\text{pig}}}} } \right)$$

Single species scaling using LBF was proposed by Ward and Smith (2004). LBF [mL/min/kg] values per kg BW were used, as BW was already incorporated into the equation with the allometric exponent of 1.0. As there was no value for pig LBF in the original paper, the LBF values were set to 4.986 mL/min/kg for humans and 20.286 mL/min/kg for pig,s respectively, as provided by Hall et al. (2012). To be used in Eq. 4, LBF_human_ and LBF_pig_ values were divided by the corresponding BW provided in the paper.

Finally, using the scaling method selected for tramadol prediction in humans, simulations of i.v. human dosing for U-47700 were performed in NONMEM®. For this purpose, the serum concentration–time profile for an individual with a BW of 70 kg given an i.v. bolus dose of 100 µg/kg was simulated 1000 times.

## Results

### Method validation

Most of the validation parameters were in the acceptable range according to the guidelines of the GTFCh (Peters et al. 2009). Detailed results of the validation and a discussion are given in the ESM (S2 and S3).

### Concentration–time profiles

After single i.v. administration, mean maximum concentrations (C_max_) in serum of 103 ± 26 ng/mL U-47700 and 802 ± 329 ng/mL tramadol were reached immediately (t = 1 min) (Fig. S1). The drug concentrations rapidly decreased within the first hour. After 60 min, mean concentrations of 17 ± 5 ng/mL U-47700 and 141 ± 34 ng/mL tramadol were found. Thereafter, the concentrations decreased slowly. At the end of the experiment, 8 h after administration, concentrations (C_last_) of 0.8 ± 0.3 ng/mL U-47700 and 6.6 ± 2.3 ng/mL tramadol were still determined in serum samples.

C_max_ values measured in whole blood samples amounted to 86 ± 23 ng/mL U-47700 and 936 ± 295 ng/mL tramadol (Fig. S2). Showing a similar concentration decrease, C_last_ values of 0.4 ± 0.3 ng/mL for U-47700 and 4.6 ± 3.4 ng/mL for tramadol were reached after 8 h.

**Fig. 1:**
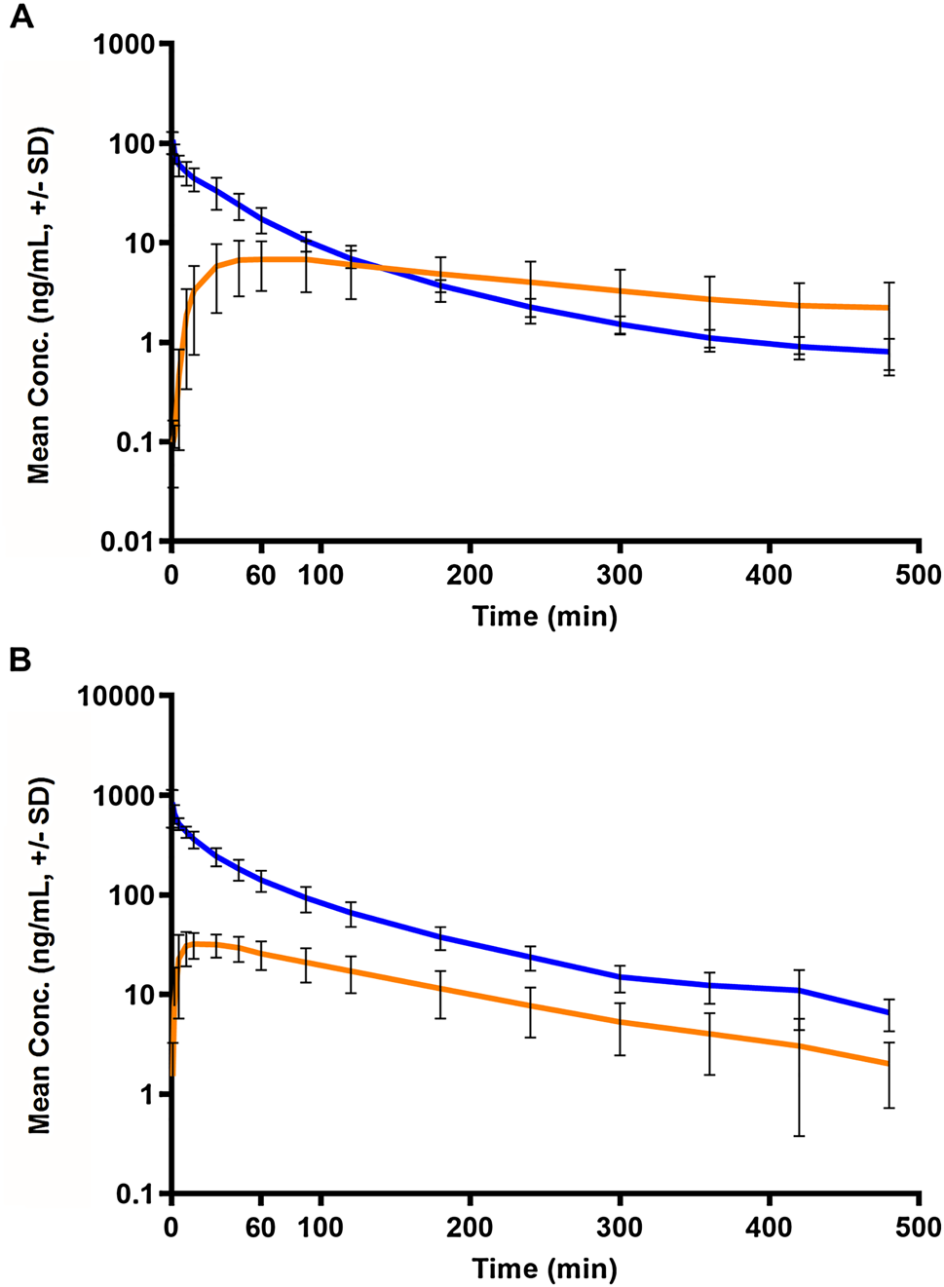
Semi-logarithmic plot of the mean concentration–time profiles including standard deviation (SD) of **A** U-47700 (blue line) and *N*-desmethyl-U-47700 (orange line) after single i.v. administration of a 100 µg/kg body weight (BW) dose, and **B** Tramadol (blue line) and *O*-desmethyltramadol (orange line) after single i.v. administration of a 1000 µg/kg BW dose determined in pig serum

In Fig. 1, the mean drug concentration–time profiles of U-47700 and tramadol in serum after single i.v. administration are plotted on a semi-logarithmic scale. The plot for U-47700 as well as for tramadol indicated a triphasic decline. These phases consisted of a tissue distribution (α) phase, an elimination (β) phase, and a tissue release (γ) phase. Half-lives (t_1/2_) for the α phase were 5.6 min for U-47700 and 6.8 min for tramadol. Calculated t_1/2_ for the β phase were 37.6 min for U-47700 and 49.3 min for tramadol. For the γ phase, t_1/2_ of 136.7 min for U-47700 and 115.7 min for tramadol could be determined. T_1/2_ of U-47700 and tramadol calculated in whole blood were in the same range for all three phases. The mean drug concentration–time profiles in whole blood are shown in Fig. S3.

C_max_ of *N*-desmethyl-U-47700 was 6.8 ± 3.2 ng/mL (Fig. S1) in serum samples and 5.0 ± 2.2 ng/mL in whole blood samples (Fig. S2) and was reached after 60 min. Afterward, the concentration of this metabolite slowly decreased in a monophasic decline. Based on this, the calculated t_1/2_ was 214 min in serum and 250 min in whole blood.

As for ODT a mean C_max_ of 32 ± 8 ng/mL could be determined 15 min after administration in serum samples (Fig. S1) and a C_max_ of 38 ± 10 ng/mL after 30 min in whole blood (Fig. S2). ODT concentrations decreased slowly afterward in a monophasic decline, as well. The calculated t_1/2_ was 103 min in serum and 107 min in whole blood.

### PopTK model development for U-47700 and tramadol

**Fig. 2:**
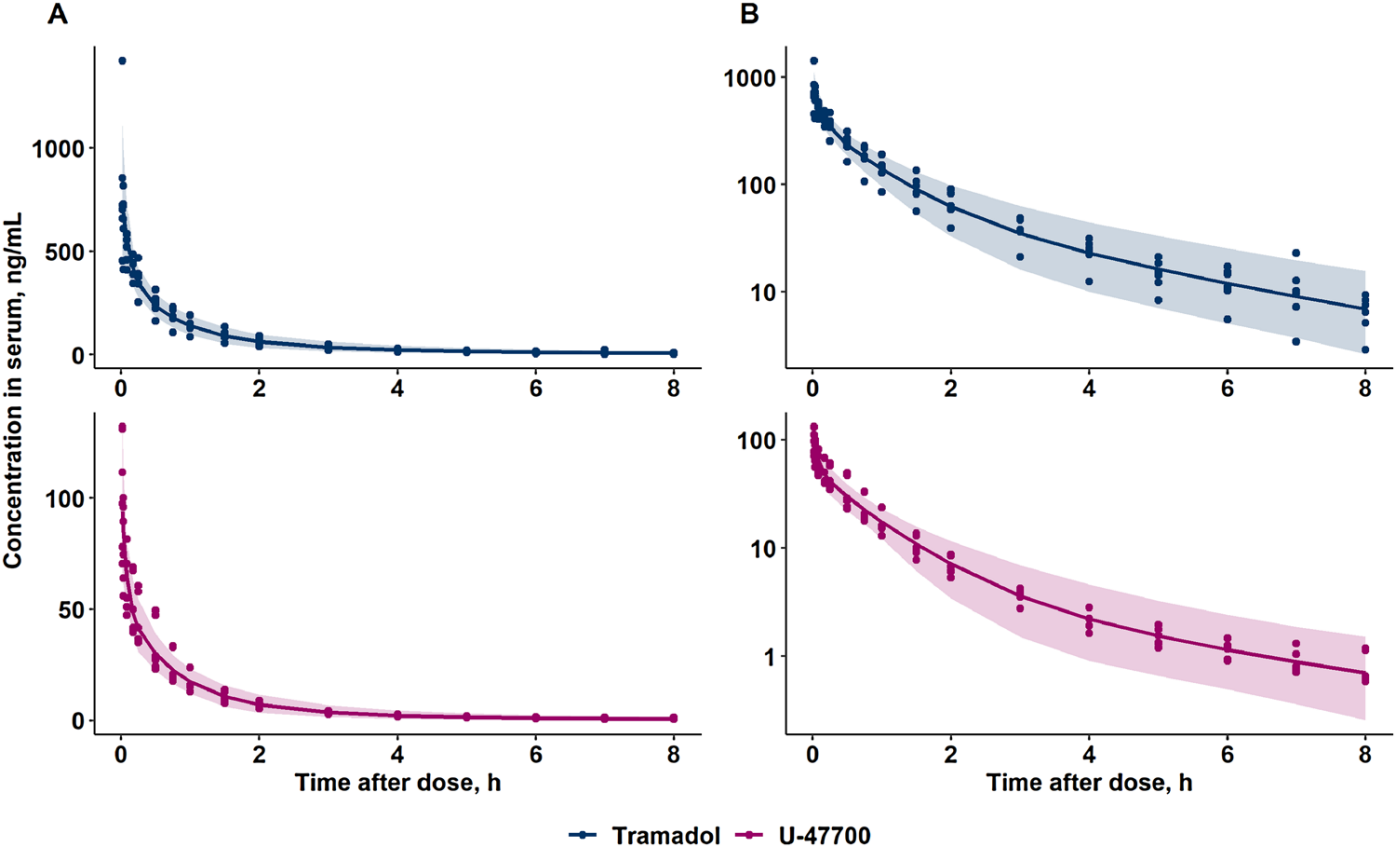
Visual predictive checks (VPCs) of final popTK models for tramadol (upper panel) and U-47700 (lower panel) on the **A** linear and **B** semi-logarithmic scale. The dots represent observed concentrations. The lines depict the median of the predicted concentrations and the shaded area is the 90% confidence interval of the predictions after 1000 simulations

During the model development process, one-, two- and three-compartment models with linear and Michaelis–Menten elimination kinetics were tested. A three-compartment model with first-order elimination processes described the data of both tramadol and U-47700 in serum the best. Model parameters in pig serum were precisely estimated with reasonable residual standard errors (RSEs) (Table 1). GOF plots (Fig. S4) and VPCs (Fig. 2) for both tramadol and U-47700 revealed no significant trend and were in good agreement with the observed data. Moderate IIV was identified on clearances (CL, Q2) and volumes of distribution (V_central_, V2, and V3) parameters as shown in Table 1. The differential equations and parameter calculations are provided in the ESM (S4). Individual TK profiles (*n* = 6) for tramadol and U-47700 can also be found in the ESM (Fig. S5).

**Table 1:** Toxicokinetic parameters of tramadol and U-47700 estimated in serum from a three-compartment pig model

Parameter	Unit	Tramadol		U-47700	
		Estimate	RSE (%)	Estimate	RSE (%)
*Structural model parameters*					
V_central_	(L/kg)	1.3	17.8	0.9	15.5
CL	(L/h/kg)	1.9	7.6	1.6	7.9
V2	(L/kg)	1.7	17.3	1.5	19.7
Q2	(L/h/kg)	0.7	13.9	0.4	18.0
V3	(L/kg)	0.7	18.9	0.6	19.0
Q3	(L/h/kg)	3.4	51.6	5.2	44.7
*Variability*					
IIV V_central_	(%CV)	27.9	33.8	24.8	9.5
IIV CL	(%CV)	16.6	31.9	19.2	15.5
IIV V2	(%CV)	30.5	49.6	54.0	33.9
IIV Q2	(%CV)	31.5	32.0	28.2	13.9
IIV V3	(%CV)	n.a	n.a	47.7	25.4
IIV Q3	(%CV)	n.a	n.a	n.a	n.a
Proportional residual error	(%)	15.0	17.8	8.5	8.1

*RSE* relative standard error, *CL* clearance from central compartment, *V* volumes of distribution, *Q* intercompartmental clearance, *IIV* interindividual variability, *CV* coefficient of variation, *n.a* not applicable

### Prediction of human tramadol and U-47700 exposure

**Fig. 3:**
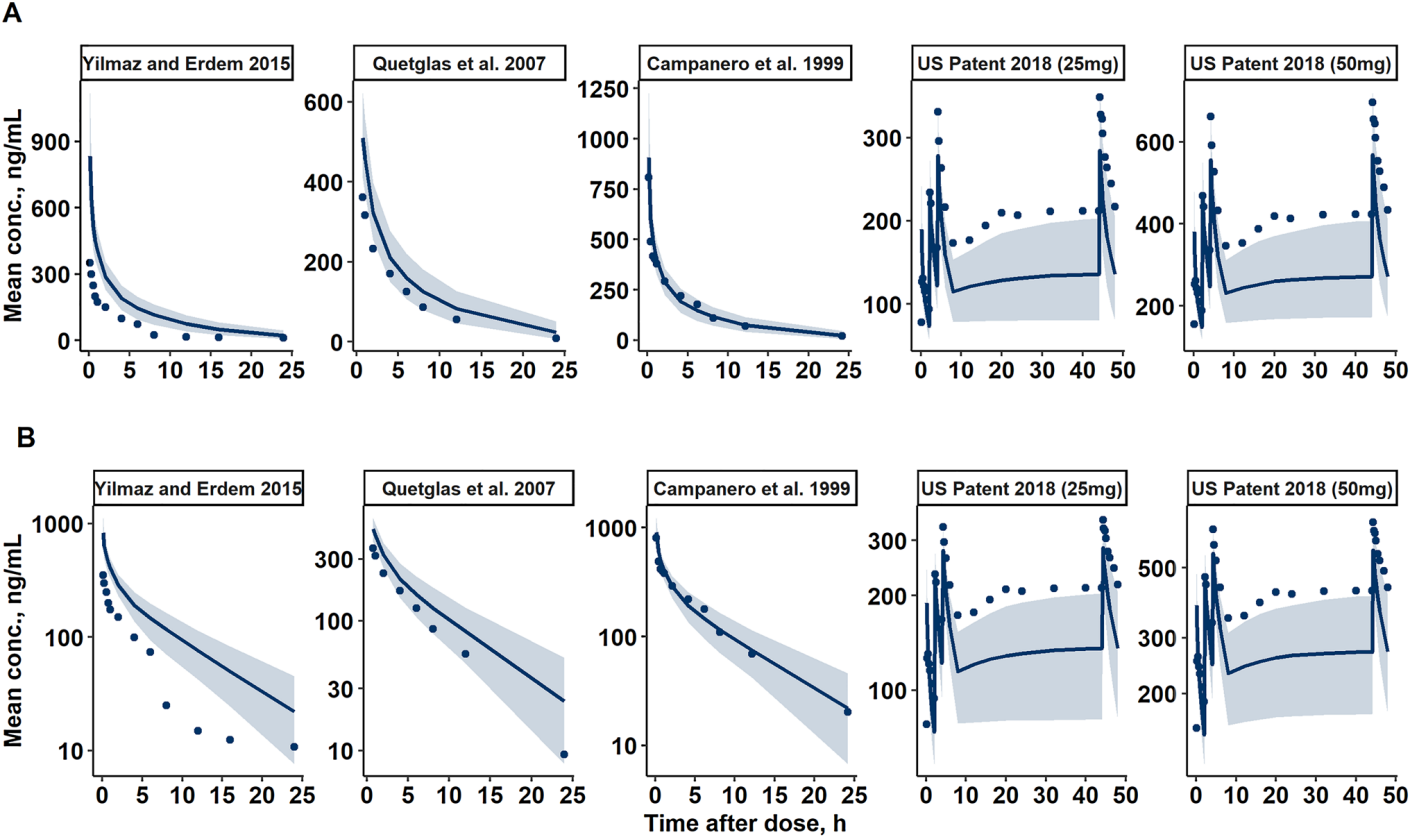
Prediction of human tramadol concentration–time profiles on the **A** linear and **B** semi-logarithmic scale. Simulated median and 90% confidence interval of the predictions after 1000 simulations shown as a solid line and shaded area respectively. Observed values digitized from literature 1–5 (Table S5) are displayed as dots

The human tramadol exposure was predicted using the final popTK pig model and different scaling techniques, described above. Multispecies allometric scaling failed to adequately predict human data, as it became clear that the interspecies variability in metabolic clearance is too large and this parameter cannot be scaled appropriately using only BW as an exponential covariate (Fig. S6). Adding the further correction factors on CL failed to sufficiently improve the situation. Using simple single species (pig, BW only) allometric scaling resulted in the substantial underprediction of the human tramadol profiles. Thus, the human tramadol exposure was best predicted using the final popTK model parameters for pigs upscaled via allometric scaling technique, which incorporated BW as an exponential covariate for all model parameters and LBF as a correction factor on metabolic clearance. The allometry exponent of 1.0 on each TK parameter described the data best and was therefore incorporated. For all five human studies (Table S5) (Campanero et al. 1999; Quetglas et al. 2007; Yılmaz and Erdem 2015; United States Patent 2018), the concentration–time profiles were predicted adequately (Fig. 3). Observed data points were mostly within the 90% prediction interval for profiles 2 and 3, slightly underpredicted for profiles 4 and 5, and slightly overpredicted for profile 1.

**Fig. 4:**
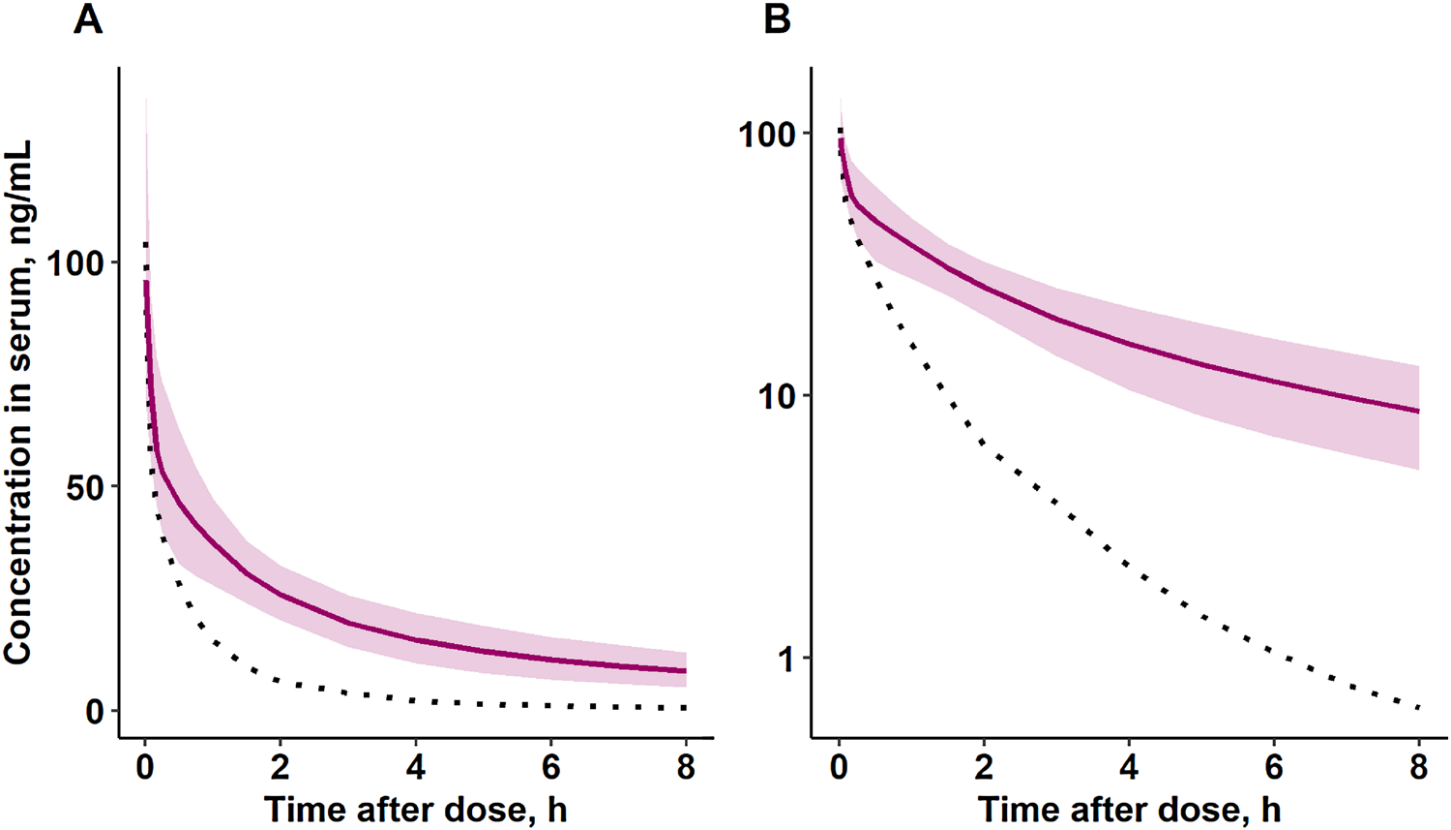
Simulation of human U-47700 serum concentration–time profile (i.v. bolus dose of 100 µg/kg, BW = 70 kg) on the **A** linear and **B** semi-logarithmic scale. Simulated median and 90% confidence interval of the predictions after 1000 simulations shown as a solid line and shaded area respectively. The dotted line shows the median U-47700 serum concentration in pig, given the same dose (100 µg/kg, i.v bolus)

The scaling technique that was considered best for tramadol was adopted for U-47700 and simulation of an i.v. bolus dose of 100 µg/kg of U-47700 for a human with BW of 70 kg was performed as shown in Fig. 4. No human profiles for U-47700 were available from the literature and therefore these predictions could not be verified with observed data.

## Discussion

### Dosage

All animals received an i.v. dose of 100 µg/kg BW U-47700 or 1000 µg/kg BW tramadol, respectively. Hence, total doses of 4.2 ± 0.1 mg of U-47700 and 42.8 ± 2.1 mg of tramadol were reached. As already described in a previous study (Nordmeier et al. 2021), the tramadol dose was similar to common human therapeutic i.v. dosages (Gelbe Liste 2020). A common (low) users’ dosage of U-47700 for the i.v. administration is about 3–5 mg (NeuePsychoaktiveSubstanzen 2017). In this context, a similar net dose was chosen for this TK study. Thereby, it was assured that the animals remained under the influence of measurable concentrations. On the other hand, they should not be exposed to great toxic effects.

### Concentration–time profiles

#### U-47700 and *N*-desmethyl-U-47700

Regarding U-47700, TK data are sparsely available. Yet, only one controlled animal study (Truver et al. 2020) was performed and one case report provides human TK data of this substance in serum (Koch et al. 2018).

Our results are in good agreement with those of Truver et al. (2020). The authors administered U-47700 (0.3, 1.0 or 3.0 mg/kg BW, *n* = 6 each) subcutaneously to male Sprague–Dawley rats. Sampling occurred from 15 until 480 min post-injection. C_max_ levels rose linearly from 40, 110 to 173 ng/mL for the different doses. Thus, they were in the same range as our C_max_ values considering the given doses. The calculated t_1/2_ were comparable as well.

Koch et al. (2018) reported the case of a 24-year-old man who suffered apnea after consumption of U-47700 in combination with the benzodiazepine flubromazepam. During hospitalization, a serum concentration of 370 ng/mL was detected 42 min after the admission to the hospital. In comparison, in our study, a maximum serum concentration of 103 ± 26 ng/mL was reached immediately after administration. This discrepancy could be a hint at a much higher dosage in the authentic case. Koch et al. (2018) estimated two elimination phases by visual inspection of the semi-logarithmic concentration–time profile of U-47700. Yet, we estimated a three-phasic decline. Koch et al. (2018) calculated a *t*_1/2_ of approximately 6 h for the first phase, which is comparable to that of morphine. *T*_1/2_ calculated in the present study were in the range of minutes for the distribution phase and up to one-half hour for the elimination phase. *T*_1/2_ of the tissue release phase was about 2.3 h and therefore differs from the values calculated by Koch et al. (2018). These high deviations may be explained by the fact that Koch et al. (2018) obtained the first serum samples 42 min after admission to the hospital. Especially, the unknown period of time between consumption and the first sampling could have a great impact on the estimated TK data. In particular, Koch et al. (2018) did not consider the first decline of U-47700. The following sampling extended up to 81 h post-admission. Hence, also late phase TK data of U-47700 were included, whereas in our study the sampling time was only up to 8 h. The TK parameter C_max_ is dose-dependent and concerning linear TK, assessed in the present study as well as by Koch et al. (2018), it is proportional to the dosage. However, in terms of linear TK, dose and concentration dependences do not apply to t_1/2_. Another issue that has to be considered in this context is that the route of administration in that case report was unknown, which could have a great impact on the TK.

TK parameters for the main metabolite *N*-desmethyl-U-47700 are only sparsely available in the literature, as well. In our study, the level of metabolite showed a medium increase with a C_max_ 60 min after drug administration. Subsequently, the concentration slowly decreased, which led to slightly higher metabolite concentrations and a higher *t*_1/2_ in comparison to U-47700 at the end of the experiment. This behavior indicates a high metabolism rate of U-47700 to *N*-desmethyl-U-47700, but a slow distribution/elimination of the metabolite. In the study of Truver et al. (2020), a slower kinetic for *N*-desmethyl-47700 was observed, as well. However, a longer *t*_1/2_ of *N*-desmethyl-U-47700 compared to U-47700 makes this metabolite interesting as a target for routine blood analysis, especially if a long time period between consumption and analysis has to be considered.

#### Tramadol and ODT

The concentration–time profile of tramadol observed in this study is in good agreement with that found by Bortolami et al. (2015), administering tramadol i.v. to sheep. They observed higher maximum serum concentrations in comparison with our values, but this difference may have resulted from higher dosages. In agreement with our data, tramadol serum concentrations decreased rapidly, but in contrast to our study, tramadol could not anymore be detected in every treated sheep 6 h following administration perhaps due to limitations of the analytical method used in that case (LOD = 5 ng/mL). The concentration–time profile for ODT was comparable as well.

Moreover, our estimated TK parameters were partly in the same range as those determined after single controlled intramuscular (i.m.) administration of tramadol to piglets (Vullo et al. 2014). Higher dosage and a different route of administration may explain some deviations mainly concerning C_max_. Considering the dose administered, the relative C_max_ determined in our study was much higher as compared to the values by Vullo et al. (2014). In both studies, high ODT levels were found over a short time period after administration. In comparison with other animals, such as dogs and goats, piglets and pigs formed higher levels of the active metabolite (Vullo et al. 2014). These differences might be due to variations in drug metabolism between different species (Martignoni et al. 2006). As ODT is an active metabolite with a higher affinity to the µ-opioid receptor, a faster metabolism in pigs and piglets has great toxicodynamic importance.

Generally, values for t_1/2_ of tramadol appeared to be very species-related and, thus, indicate differences in metabolism and TK in different species as well. The *t*_1/2,γ_ of tramadol observed in this study (1.9 h) was a little bit higher than terminal *t*_1/2_ reported for piglets (1.34 h) (Vullo et al. 2014), horses (1.5 h) (Shilo et al. 2008), and dogs (0.73 h) (Giorgi et al. 2010), but lower than that reported in llama (2.54 h) (Cox et al. 2011). Regarding the human terminal t_1/2_ of tramadol published in the literature (Murthy et al. 2000; Grond and Sablotzki 2004; World Health Organization 2014), values between 5 and 6 h were mostly determined. In the present study, a quite shorter terminal *t*_1/2_ of about 2 h was estimated. Of course, a faster metabolization and elimination of tramadol may have a big influence on this parameter. Another explanation for this possible underestimation is the much shorter sampling period of 8 h in our study as compared to other studies covering a sampling period up to 48–72 h (Meyer et al. 2015; Skinner-Robertson et al. 2015; DeLemos et al. 2017). Thus, the *t*_1/2_ of this study only reflects the elimination within the first 8 h following administration, and the real terminal t_1/2_ could only be estimated in a long-term elimination study.

Regarding human studies, the concentration–time profile of our study was in good agreement with that of Murthy et al. (2000), administering a 2 mg/kg i.v. dose to children. However, in our study, a faster increase in the metabolite concentration was observed. Maximum concentrations were reached after 15 min, whereas Murthy et al. (2000) observed maximum concentrations 4.9 h following the administration. A slower metabolism in humans than in animals has already been reported in the literature (Grond and Sablotzki 2004) and our results indicate a faster metabolization in pigs as well. In our study, estimated TK parameters as V and CL also differ from those calculated in those human studies (Murthy et al. 2000). As already shown in other animal studies, CL values of pigs were higher than those obtained for humans after i.v. administration, supporting the assumption of a faster metabolism of tramadol in pigs compared to human (Grond and Sablotzki 2004; Vullo et al. 2014). On the other hand, in humans, a higher V (~ 3L/kg) was observed as compared to our study (V =  ~ 1.25 L/kg) indicating a higher distribution in deep compartments (Murthy et al. 2000).

Comparing C_max_ values of tramadol after i.v. administration reported in the literature with our results substantially lower substance plasma concentrations of about 400 ng/mL were determined in studies using comparable dosages (100 mg/ ~ 70 kg for humans vs. 1 mg/kg in our study) (Lintz et al. 1986, 1998a,b). However, differences in analytics in serum and plasma need to be taken into consideration comparing serum and plasma concentrations. Plasma could be contaminated with cells, lysed cells could release their content into plasma or cells could be still metabolically active in plasma (Bowen et al. 2010). These processes might have an influence on the compound concentration determined in plasma and could result in higher concentrations if substances have a prevalence for binding to blood cells or it could affect the ratio of the concentrations of the parent compound to metabolite. Furthermore, in humans a higher V (~ 3L/kg) (World Health Organization 2014) was determined as compared to pigs (~ 1.25 L/kg). A higher tissue affinity and distribution into deep compartments result in lower blood concentrations of tramadol and therefore could be an explanation for this phenomenon.

### General findings and TK parameters of U-47700 and tramadol

The choice of the matrix (plasma, serum, or whole blood) is an important aspect of the drug quantification for the TK/PK studies. Currently, the majority of the PK studies are using plasma (or serum) as a matrix of choice rather than whole blood. Concerning drugs with high binding to erythrocytes, this difference can influence some TK parameters (e.g., increased volume of distribution), as less drug is left in the plasma compared to the whole blood (Dash et al. 2020). For this reason, in the current study both serum and whole blood were used as matrices. In general, the concentration–time profiles of U-47700 and its metabolite in serum and whole blood illustrated that the determined concentrations in serum samples were higher than those in whole blood samples, leading to the suggestion that U-47700 and its metabolite might not accumulate in red blood cells. In contrast, tramadol and ODT generally displayed lower concentrations in serum than in whole blood, indicating the binding of those substances to red blood cells. Some literature data are available signifying the formation of tramadol complexes with human hemoglobin (Tunç et al. 2013), but its relevance for the tramadol PK remains unclear.

In misuse and poisoning cases in clinical and forensic toxicology, serum samples are the matrix of choice for analytics, and serum TK data are of special interest whereas plasma TK data are of minor interest. Hence, the TK study was performed with serum and whole blood samples and not with plasma usually chosen for PK/TK studies. Since tramadol PK studies have almost exclusively used plasma as a matrix rather than whole blood, the popTK modeling and simulations were only performed for serum data of the parent compounds. Thus, the possibility of a slight difference between plasma and serum concentrations must be considered, when interpreting the modeling results.

The anesthesia of the animals was performed using ketamine/xylazine and maintained with isoflurane. In general, these substances might have an impact on substance and metabolite concentrations of simultaneously administered drugs. However, xylazine and ketamine have short t_1/2_ (xylazine: 2.8–5.4 min following intramuscular administration; ketamine: 11 min following i.v. administration) and were administered before all chirurgical procedures (Garcia-Villar et al. 1981; Wieber et al. 1975). Together with the fact of some preparation time before the start of the TK experiment, interactions of these substances with the opioids of interest should be negligible. For the further anesthesia, isoflurane was chosen instead of thiopental, a common anesthetic, to avoid possible CYP interaction (Bovill 1997). Isoflurane is predominantly metabolized by CYP2E1 (Kharasch et al. 1999), whereas U-47700 is mainly metabolized through CYP2B6, CYP2C19 and CYP3A4 (Nordmeier et al. 2020a) and tramadol by CYP2D6 and CYP3A4 (DeLemos et al. 2017). Thus, significant interactions of isoflurane with the TK were not expected.

The substance administration and blood sampling occurred via a triple lumen central venous catheter. Hence, a contamination of the blood samples with substance residues in the administration lumen could be excluded due to the spatial separation. Sampling occurred above the bloodstream. Following administration, the substance was rapidly distributed via the bloodstream. Thus, an influence on the determined analyte concentrations by the localization of application and sampling, also shortly after administration, seems unlikely. In general, our TK parameters were comparable with those of Truver et al. (2020) administering U-47700 subcutaneously to rats and concentration–time profiles of tramadol were comparable to literature data addressing i.v. administration as well (Bortolami et al. 2015). Hence, only a minor influence on determined concentrations by the localization of the application and sampling site is likely. Furthermore, the general influence of a central sampling site in combination with a central application should be addressed. Due to this route of application, an accumulation in central tissues such as the heart is possible. This issue might play a major role in postmortem studies and tissue distribution, but should play a minor role in living organisms with a fast blood circulation. In this setting, only minor deviations between a central and peripheral sampling are expected.

TK parameters of U-47700 and tramadol determined in pig serum showed similar behavior. In both cases, the three-compartment model described the data best. Central CL was estimated to be 1.9 L/h/kg and 1.6 L/h/kg for tramadol and U-47700, respectively, indicating a moderate to fast elimination of both substances. The V_central_ was estimated to be 1.3 L/kg for tramadol and 0.9 L/kg for U-47700. The determined V values of both substances indicate a slight distribution into deep compartments. The smaller V_central_ values compared to the first peripheral V2 suggest a rapid distribution from the central compartment into highly perfused tissues, whereas the higher V_central_ values compared to the second peripheral V3 suggest a slower distribution into deep compartments (scarcely perfused tissues). Thus, higher concentrations of both substances in different tissues as compared to blood concentrations are possible regarding postmortem toxicological cases. Furthermore, an accumulation in adipose tissue has to be taken into consideration (Nordmeier et al. 2021).

In general, estimated TK parameters for tramadol tend to be very similar, but slightly higher than for U-47700.

### Prediction of human tramadol exposure

To predict the human exposure of drugs from animal studies, the allometric scaling technique is commonly used. This method is an empirical approach that is based on the assumption of the similarities in the physiology across different species and correlates physiological parameters, such as CL and V with body size using a power function. The method is widely used in drug development (e.g., first in human studies) (Hunter 2010).

In the current analysis both multiple and single (pig) species allometry scalings were attempted. Multiple species allometry (Fig. S6) was challenging, as the interspecies variability in metabolic clearance between species, as well as in humans was very large despite adjusting for the differences in BW. This can be attributed to the interspecies differences in the expression of CYPs, which are the most important family of drug-metabolizing enzymes and the major cause of species differences in hepatic drug metabolism (Martignoni et al. 2006). Though additional correction factors (MLP, BRW, LBF, bile flow, and LW) were tested to address this issue, they either did not improve the prediction or were only available for some of the species. Due to these issues, we have proceeded with single (pig) species allometry (Fig. 3).

An empirical allometric exponent for the majority of the small-molecule drugs lay between 0.4 and 1.5, dependent on the elimination pathway (renal, hepatic, or mixed) and characteristic of the drug (Huh et al. 2011). Huh et al. (2011) reported no value for tramadol, but substances that have similar elimination and characteristics as tramadol (base, hepatic/mixed elimination) had an exponent value around 0.7–0.8 (e.g., 0.805 for fentanyl, 0.775 for methadone). Huang et al. (2015) reported the allometric exponent b for tramadol to be around 1.14 (based on the linear regressions of the log‐log transformed clearance vs. BW data). For the current analysis, allometric exponents between 0.5 and 1.6 were tested, and b = 1.0 was found to fit the best (evaluated using dOFV, %RSE, and GOF plots). This value is close to the values found in the literature for tramadol and similar substances.

The simple allometric scaling generally works well for small-molecule drugs that are mainly renally eliminated. However, for hepatically eliminated drugs with a large between-species variability in hepatic metabolism, simple allometric scaling may not work very well in the extrapolation of hepatic metabolic CL from animals to humans. Thus, to improve the predictions, liver metabolism should be incorporated as well as BW (Huh et al. 2011). This seems to be the case for tramadol, as it is primarily eliminated through metabolism by the liver (by CYP2D6, CYP3A4, and CYP2B6 in humans). The incorporation of the LBF on CL significantly improved human predictions for tramadol. LBF was previously reported to be directly proportional to liver weight in the majority of the mammalian species. Accordingly, liver weight (and therefore hepatic blood flow) could be related to BW (Boxenbaum 1980). In humans, LBF depends not only on liver and BW, but also changes with age and with disease progression in chronic liver disease, which make extrapolation of hepatic metabolic CL from animals to humans even more challenging (Woodhouse and Wynne 1988). Thus, the current analysis shows that even with the incorporation of both BW and LBF, not all profiles could have been described perfectly. The literature data used for human predictions provided further difficulties, as for three of five available profiles, no BW data were reported, so it was assumed, that BW = 70 kg. This assumption, by all means, can affect the accuracy of the prediction, if the individual/mean BW varies greatly from the assumed 70 kg. A further limitation of the current analysis lies in the availability and inconsistency of the physiological parameters used as correction factors on CL for a pig. For example, there were multiple values of LBF for pigs available in the literature: Boxenbaum et al. (1980) reported hepatic blood flow of 43.75 mL/min/kg, though the LBF_pig_ value of 20.286 mL/min/kg was provided by Hall et al. (2012). In the case of our analysis, the value that provided the best fit was used, nevertheless one should be aware of heterogeneity of the available animal data, as well as of a strain of the animal being used (e.g., different pig strains, minipig, or micropig). Nevertheless, despite some limitations, single species scaling from pig to human using LBF was shown to be able to predict human data reasonably well.

As there were a lot of similarities between tramadol and U-47700 popTK in pigs, we assumed that the same scaling method could be applied to simulate the potential concentration–time curve of U-47700 for humans. This approach applies that we also assume a faster metabolism of U-47700 in pigs compared to human, as shown in Fig. 4. The main limitation here was a lack of human TK data for U-47700 so far. Therefore, further research of human TK for U-47700 is needed to evaluate the human prediction using observed human data.

## Conclusion

In the present study, serum and whole blood concentration–time profiles were successfully determined for U-47700, tramadol, and their main metabolites in pigs after i.v. administration using a fully validated LC–MS/MS method. A three-compartment popTK model with first-order elimination described the serum concentration–time profiles for U-47700 and tramadol the best. Parameter estimates for U-47700 and tramadol are very similar but slightly higher for tramadol than for U-47700. The V_central_ indicates a slight distribution of both substances into deep compartments and central clearances a medium to fast elimination. A higher t_1/2_ of *N*-desmethyl-U-47700 compared to its parent compound U-47700 makes this metabolite interesting as a target for analytical approaches in forensic and clinical toxicology. To draw a conclusion, whilst a multiple species scaling approach failed to adequately predict human tramadol TK data, the successful prediction of human tramadol TK data based on this popTK pig model proposes that pigs in combination with a single species TK modeling technique provide a helpful tool for the prediction of human TK of NSOs. Hereby generated data might offer an enhancement in the interpretation of analytical results in clinical and forensic misuse or poisoning cases.

## Supplementary Information

Below is the link to the electronic supplementary material.

Supplementary file1 (DOCX 632 KB)

## References

[CR1] Alzghari SK, Fleming SW, Rambaran KA (2017). U-47700: An emerging threat. Cureus.

[CR2] Anzenbacher P, Soucek P, Anzenbacherova E (1998). Presence and activity of cytochrome P450 isoforms in minipig liver microsomes comparison with human liver samples. Drug Metab Dispos.

[CR3] Beal SL, Boeckmann A, Bauer RJ (2009). Nonmem user’s guides.

[CR4] Bortolami E, Della Rocca G, Di Salvo A (2015). Pharmacokinetics and antinociceptive effects of tramadol and its metabolite O-desmethyltramadol following intravenous administration in sheep. Vet J.

[CR5] Bovill JG (1997). Adverse drug interactions in anesthesia. J Clin Anesth.

[CR6] Bowen RAR, Hortin GL, Csako G (2010). Impact of blood collection devices on clinical chemistry assays. Clin Biochem.

[CR7] Boxenbaum H (1980). Interspecies variation in liver weight, hepatic blood flow, and antipyrine intrinsic clearance: Extrapolation of data to benzodiazepines and phenytoin. J Pharmacokinet Biopharm.

[CR8] Campanero MA, Calahorra B, Valle M, et al (1999) Enantiomeric separation of tramadol and its active metabolite in human plasma by chiral high-performance liquid chromatography: Application to pharmacokinetic studies. Chirality 11:272–279. 10.1002/(SICI)1520-636X(1999)11:4<272::AID-CHIR3>3.0.CO;2-I 10.1002/(SICI)1520-636X(1999)11:4<272::AID-CHIR3>3.0.CO;2-I 10224654

[CR9] Coopman V, Blanckaert P, Van Parys G, Van Calenbergh S, Cordonnier J (2016). A case of acute intoxication due to combined use of fentanyl and 3,4-dichloro-N-[2-(dimethylamino)cyclohexyl]-N-methylbenzamide (U-47700). Forensic Sci Int.

[CR10] Cox S, Martín-Jiménez T, Amstel S, Doherty T (2011). Pharmacokinetics of intravenous and intramuscular tramadol in llamas. J Vet Pharmacol Ther.

[CR11] Dash RP, Veeravalli V, Thomas JA (2020). Whole blood or plasma: what is the ideal matrix for pharmacokinetic-driven drug candidate selection?. Future Med Chem.

[CR12] DeLemos B, Richards HM, Vandenbossche J (2017). Safety, tolerability, and pharmacokinetics of therapeutic and supratherapeutic doses of tramadol hydrochloride in healthy adults: A randomized, double-blind, placebo-controlled multiple-ascending-dose study. Clin Pharmacol Drug Dev.

[CR13] EMCDDA: European Monitoring Center of Drugs and Drug Addiction (2019) European drug report – Trends and developments. http://www.emcdda.europa.eu/system/files/publications/11364/20191724_TDAT19001ENN_PDF.pdf. Accessed March 2021

[CR14] Evangelista Vaz R, Draganov DI, Rapp C (2018). Preliminary pharmacokinetics of tramadol hydrochloride after administration via different routes in male and female B6 mice. Vet Anaesth Analg.

[CR15] Fleming SW, Cooley JC, Johnson L (2017). Analysis of U-47700, a novel synthetic opioid, in human urine by LC-MS-MS and LC-QToF. J Anal Toxicol.

[CR16] Garcia-Villar R, Toutain PL, Alvinerie M, Ruckenbusch Y (1981). The pharmacokinetics of xylazine hydrochloride: an interspecific study. J Vet Pharmacol Ther.

[CR17] Giorgi M, Del Carlo S, Łebkowska-Wieruszewska B, Kowalski CJ, Saccomanni G (2010). Pharmacokinetics of tramadol and metabolites after injective administrations in dogs. Pol J Vet Sci.

[CR18] Grond S, Sablotzki A (2004). Clinical pharmacology of tramadol. Clin Pharmacokinet.

[CR19] Hall C, Lueshen E, Mošat’ A, Linninger AA,  (2012). Interspecies scaling in pharmacokinetics: A novel whole-body physiologically based modeling framework to discover drug biodistribution mechanisms in vivo. J Pharm Sci.

[CR20] Huang Q, Gehring R, Tell LA, Li M, Riviere JE (2015). Interspecies allometric meta-analysis of the comparative pharmacokinetics of 85 drugs across veterinary and laboratory animal species. J Vet Pharmacol Ther.

[CR21] Huh Y, Smith DE, Rose Feng M (2011). Interspecies scaling and prediction of human clearance: comparison of small- and macro-molecule drugs. Xenobiotica.

[CR22] Hunter R (2010). Interspecies allometric scaling. Handb Exp Pharmacol.

[CR23] Jamali B, Sheikholeslami B, Hosseinzadeh Ardakani Y (2017). Evaluation of the ecstasy influence on tramadol and its main metabolite plasma concentration in rats. Drug Metab Pers Ther.

[CR24] Kelly KR, Pypendop BH, Christe KL (2015). Pharmacokinetics of tramadol following intravenous and oral administration in male rhesus macaques (Macaca mulatta). J Vet Pharmacol Ther.

[CR25] Kharasch ED, Hankins DC, Cox K (1999). Clinical isoflurane metabolism by cytochrome P450 2E1. Anesthesiology.

[CR26] Knych HK, Corado CR, Mckemie DS, Steffey EP (2013). Pharmacokinetics and selected pharmacodynamic effects of tramadol following intravenous administration to the horse. Equine Vet J.

[CR27] Koch K, Auwaerter V, Hermanns-Clausen M, Wilde M, Neumann MA (2018). Mixed intoxication by the synthetic opioid U-47700 and the benzodiazepine flubromazepam with lethal outcome: Pharmacokinetic data. Drug Test Anal.

[CR28] Lehmann S, Teifel D, Rothschild MA, Andresen-Streichert H (2018). Tödliche Intoxikation mit dem Designer-Opioid U-47700. Tochichem Krimtech.

[CR29] Lintz W, Barth H, Osterloh G, Schmidt-Boethelt E (1986). Bioavailability of enteral tramadol formulations. 1st communication: capsules. Arzneimittelforschung.

[CR30] Lintz W, Barth H, Becker R (1998). Pharmacokinetics of tramadol and bioavailability of enteral tramadol formulations. 2nd communication: drops with ethanol. Arzneimittelforschung.

[CR31] Lintz W, Barth H, Osterloh G (1998). Pharmacokinetics of tramadol and bioavailability of enteral tramadol formulations. 43rd communication: suppositories. Arzneimittelforschung.

[CR32] Gelbe Liste (2020). Pharmindex. https://www.gelbe-liste.de/wirkstoffe/Tramadol_1406. Accessed March 2021

[CR33] Martignoni M, Groothuis GMM, de Kanter R (2006). Species differences between mouse, rat, dog, monkey and human CYP-mediated drug metabolism, inhibition and induction. Expert Opin Drug Metab Toxicol.

[CR34] Matuszewski BK, Constanzer ML, Chavez-Eng CM (2003). Strategies for the assessment of matrix effect in quantitative bioanalytical methods based on HPLC-MS/MS. Anal Chem.

[CR35] Meyer MR, Rosenborg S, Stenberg M, Beck O (2015). First report on the pharmacokinetics of tramadol and O-desmethyltramadol in exhaled breath compared to plasma and oral fluid after a single oral dose. Biochem Pharmacol.

[CR36] Mohr ALA, Friscia M, Papsun D, Kacinko SL, Buzby D, Logan BK (2016). Analysis of novel synthetic opioids U-47700, U-50488 and furanyl fentanyl by LC-MS/MS in postmortem casework. J Anal Toxicol.

[CR37] Murthy B, Pandya K, Booker P, Murray A, Lintz W, Terlinden R (2000). Pharmacokinetics of tramadol in children after IV or caudal epidural administration. Br J Anaesth.

[CR38] NeuePsychoaktiveSubstanzen (2017) http://neuepsychoaktivesubstanzen.de/u-47700/#U-47700_Dosis_Dosierung. Accessed March 2021

[CR39] Nordmeier F, Doerr A, Laschke MW (2020). Erhebung toxikokinetischer Daten der synthetischen Opioide U-47700 und Tramadol sowie der Hauptmetabolite im Schwein nach intravenöser Verabreichung. 99. Jahrestagung der Deutschen Gesellschaft für Rechtsmedizin. Abstracts Rechtsmedizin.

[CR40] Nordmeier F, Doerr A, Laschke MW (2020). Are pigs a suitable animal model for in vivo metabolism studies of new psychoactive substances? A comparison study using different in vitro/in vivo tools and U-47700 as model drug. Toxicol Lett.

[CR41] Nordmeier F, Doerr AA, Potente S (2021). Perimortem distribution of U-47700, tramadol and their main metabolites in pigs following intravenous administration. J Anal Toxicol.

[CR42] Peters FT, Drummer OH, Musshoff F (2007). Validation of new methods. Forensic Sci Int.

[CR43] Peters F, Paul L, Musshoff F, et al (2009) Anhang B zur Richtlinie der GTFCh zur Qualitätssicherung bei forensisch-toxikologischen Untersuchungen Anforderungen an die Validierung von Analysemethoden. Toxichem Krimtech. https://www.gtfch.org/cms/images/stories/files/GTFCh_Richtlinie_Anhang B_Validierung_Version 1.pdf. Accessed March 2021

[CR44] Puccinelli E, Gervasi P, Longo V (2011). Xenobiotic metabolizing cytochrome P450 in pig, a promising animal model. Curr Drug Metab.

[CR45] Quetglas EG, Azanza JR, Cardenas E (2007). Stereoselective pharmacokinetic analysis of tramadol and its main phase I metabolites in healthy subjects after intravenous and oral administration of racemic tramadol. Biopharm Drug Dispos.

[CR46] R.C. Team (2018). R: A language and environment for statistical computing.

[CR47] Rambaran KA, Fleming SW, An J (2017). U-47700: A Clinical Review of the Literature. J Emerg Med.

[CR48] Rohrig TP, Miller SA, Baird TR (2017). U-47700: A not so new opioid. J Anal Toxicol.

[CR49] Schaefer N, Kettner M, Laschke MW (2015). Simultaneous LC-MS/MS determination of JWH-210, RCS-4, ∆9-tetrahydrocannabinol, and their main metabolites in pig and human serum, whole blood, and urine for comparing pharmacokinetic data. Anal Bioanal Chem.

[CR50] Schaefer N, Wojtyniak J-G, Kettner M (2016). Pharmacokinetics of (synthetic) cannabinoids in pigs and their relevance for clinical and forensic toxicology. Toxicol Lett.

[CR51] Schaefer N, Kettner M, Laschke MW (2017). Distribution of synthetic cannabinoids JWH-210, RCS-4 and Δ 9-tetrahydrocannabinol after intravenous administration to pigs. Curr Neuropharmacol.

[CR52] Schaefer N, Wojtyniak J-G, Kroell A-K (2018). Can toxicokinetics of (synthetic) cannabinoids in pigs after pulmonary administration be upscaled to humans by allometric techniques?. Biochem Pharmacol.

[CR53] Schaefer N, Kroell A-K, Koerbel C (2020). Time- and temperature-dependent postmortem concentration changes of the (synthetic) cannabinoids JWH-210, RCS-4, as well as ∆9-tetrahydrocannabinol following pulmonary administration to pigs. Arch Toxicol.

[CR54] Shilo Y, Britzi M, Eytan B, Lifschitz T, Soback S, Steinmann A (2008). Pharmacokinetics of tramadol in horses after intravenous, intramuscular and oral administration. J Vet Pharmacol Ther.

[CR55] Skinner-Robertson S, Fradette C, Bouchard S, Mouksassi MS, Varin F (2015). Pharmacokinetics of tramadol and O-desmethyltramadol enantiomers following administration of extended-release tablets to elderly and young subjects. Drugs Aging.

[CR56] Solimini R, Pichini S, Pacifici R, Busardò FP, Giorgetti R (2018). Pharmacotoxicology of non-fentanyl derived new synthetic opioids. Front Pharmacol.

[CR57] Truver MT, Smith CR, Garibay N, Kopajtic TA, Swortwood MJ, Baumann MH (2020). Pharmacodynamics and pharmacokinetics of the novel synthetic opioid, U-47700, in male rats. Neuropharmacology.

[CR58] Tunç S, Çetinkaya A, Duman O (2013). Spectroscopic investigations of the interactions of tramadol hydrochloride and 5-azacytidine drugs with human serum albumin and human hemoglobin proteins. J Photochem Photobiol B Biol.

[CR59] United States Patent (2018). Intravenous administration of tramadol. US 10,022, 321 B2. Accessed March 2021.

[CR60] Vullo C, Kim TW, Meligrana M, Marini C, Giorgi M (2014). Pharmacokinetics of tramadol and its major metabolite after intramuscular administration in piglets. J Vet Pharmacol Ther.

[CR61] Ward KW, Smith BR (2004). A comprehensive quantitative and qualitative evaluation of extrapolation of intravenous pharmacokinetic parameters from rat, dog, and monkey to humans. I Clearance Drug Metab Dispos.

[CR62] Wickham H (2009). ggplot2: Elegant Graphics for Data Analysis.

[CR63] Wieber J, Gugler R, Hengstmann JH, Dengler HJ (1975). Pharmacokinetics of ketamine in man. Anaesthesist.

[CR64] Woodhouse KW, Wynne HA (1988). Age-related changes in liver size and hepatic blood flow. Clin Pharmacokinet.

[CR65] World Health Organization (2014), Expert committee on drug dependence thirty‐sixth meeting. Tramado l update review report agenda item 6.1. https://www.who.int/medicines/areas/quality_safety/6_1_Update.pdf. Accessed March 2021

[CR66] World Health Organization (2016) Expert committee on drug dependence thirty-eighth meeting. U-47700 critical review report agenda item 4.1. https://www.who.int/medicines/access/controlled-substances/4.1_U-47700_CritReview.pdf?ua=1. Accessed March 2021

[CR67] Yılmaz B, Erdem AF (2015). Simultaneous determination of tramadol and its metabolite in human Plasma by GC/MS. J AOAC Int.

