# Supplementary material for: Toxicokinetics of U-47700, tramadol, and their main metabolites in pigs following intravenous administration: is a multiple species allometric scaling approach useful for the extrapolation of toxicokinetic parameters to humans?
Source: Arch Toxicol. 2021 Oct 3;95(12):3681–93. doi: 10.1007/s00204-021-03169-y (PMC8536616; doi:10.1007/s00204-021-03169-y)
Supplement: Supplementary file 1 — Supplementary file1 (DOCX 632 KB) [file 204_2021_3169_MOESM1_ESM.docx]

**Electronic Supplementary Material**

**Toxicokinetics of U-47700, tramadol, and their main metabolites in pigs following intravenous administration – Is a multiple species allometric scaling approach useful for the extrapolation of toxicokinetic parameters to humans?**

Frederike Nordmeier^1^, Iryna Sihinevich^2^, Adrian A. Doerr^1^, Nadja Walle^1^, Matthias W. Laschke^3^, Thorsten Lehr^2^, Michael D. Menger^3^, Peter H. Schmidt^1^, Markus R. Meyer^4^, and Nadine Schaefer^1*^

^1^Institute of Legal Medicine, Saarland University, 66421 Homburg, Germany

^2^Clinical Pharmacy, Saarland University, 66123 Saarbruecken, Germany

^3^Institute for Clinical and Experimental Surgery, Saarland University, 66421 Homburg, Germany

^4^Department of Experimental and Clinical Toxicology, Institute of Experimental and Clinical Pharmacology and Toxicology, Center for Molecular Signaling (PZMS), Saarland University, 66421 Homburg, Germany

**S1 Methods**

**Chemicals and reagents**

Hydrochloric acid, di-potassium hydrogen phosphate, ammonia solution 25% EMSURE, potassium hydroxide, and formic acid EMSURE were purchased from Merck (Darmstadt, Germany). Na_2_EDTA, dichloromethane, and ammonium formate were obtained from Sigma-Aldrich (Steinheim, Germany). From Fisher chemicals (Loughborough, UK) HPLC-grade water, acetonitrile, methanol, and ethanol were bought. Standards as *N*-desmethyl-U-47700 (solid), methanolic solutions of dehydrocodeine, fentanyl, norfentanyl, morphine (0.1 mg/mL each), tramadol-HCl, *O*-desmethyltramadol-HCl (ODT), codeine, EDDP, hydromorphone, hydrocodone, 6-monoacetylmorphine (6-MAM), methadone, oxycodone, tilidine, and nortilidine (1 mg/mL each), U-51754 hydrochloride (solid), U-47931E (solid), methoxyacetylfentanyl hydrochloride (solid), tramadol-HCl-*C*_13_-*d*_3_ (1 mg/mL), and ODT-*d*_6_ (0.1 mg/mL) were from LGC (Wesel, Germany). *N*-desmethyl-U-47700-*d*_3_ (0.1 mg/mL in acetonitrile) was purchased from Sigma-Aldrich (Taufkirchen, Germany). Tramadol-HCl solution used for drug administration (Tramadol Denk 100 mg in 2 mL) was bought from Denk Pharma (Munich, Germany). U-47700 hydrochloride (purity 92.6%) was offered by the German Federal Crime Police Office (Wiesbaden, Germany) for research purposes.

**Experimental preparation**

**Buffers**

For the preparation of the phosphate buffer (0.1 M, pH 6), 13.61 g di-potassium hydrogen phosphate was dissolved in 1 L deionized water. Potassium hydroxide solution (1 M) was added for pH adjustment.

**Blank serum, and whole blood samples**

Blank blood samples used for method development and validation were taken from drug-free pigs (Swabian Hall strain, Emil Faerber GmbH & Co. KG, Zweibruecken, Germany). Before freezing, blood samples were divided into two aliquots. One aliquot was centrifuged at 1250 g for 15 min to obtain serum samples. Na_2_EDTA (1.64 mg/mL) was given to the whole blood samples for prevention of clotting. All samples were stored at - 20 °C until analysis.

**Stock solutions, calibration standards, and quality control samples**

The solid compound was dissolved in methanol to generate standard stock solutions of U-47700 and *N*-desmethyl-U-47700 (1 mg/mL). Working standard solutions (0.001, 0.01, 0.1 mg/mL) were generated by the dilution of the stock solutions or liquid reference standards with ethanol, respectively. Spiking solutions for calibration standards were prepared in serum at concentrations of 0.25, 0.5, 1, 5, 10, 15, 20, 50, and 100 ng/mL, and in whole blood in concentrations of 0.5, 1, 5, 10, 15, 20, 50, and 100 ng/mL through the dilution of working solutions with ethanol. Quality control (QC) LOW, MID, and HIGH samples were prepared in the same way. QC concentrations are shown in Table S3 and S4. All solutions were stored at - 20 °C.

**Sample preparation**

According to a previous study (Nordmeier et al. 2021), solid-phase extraction (SPE) was performed with Bond Elut HCX cartridges (130 mg/3 mL; Agilent, Waldbronn, Germany). Condition of the columns was carried out by two washing steps with 3 mL methanol and one with 3 mL phosphate buffer (0.1 M, pH 6). For the validation experiments, 1 mL serum or whole blood was added to a mixture of 50 µL of an ethanolic stable-isotope-labeled internal standard mixture solution (SIL-IS; 10 ng/50 µL of *N*-desmethyl-U-47700-*d*_3_, tramadol-*C*_13_-*d*_3_, and *O*-desmethyltramadol (ODT)-*d*_6_), 50 µL spiking solution, and 2 mL phosphate buffer for validation experiments. For authentic samples, the spiking solution was replaced by ethanol. After vortexing, the samples were centrifuged at 3200 g for 8 min and loaded onto the cartridges. Subsequently, two washing steps with 2 mL phosphate buffer and 2.75 mL hydrochloric acid (0.01 M) were carried out. The columns were carefully dried with swabs and then exposed for 10 min to maximum vacuum (10 inHg). Then, they were dried another time for 3 min under maximum vacuum following the treatment with 2 mL of methanol. Analyte elution was achieved using 1.75 mL dichloromethane-methanol-ammonia solution (25%) (30:15:1.25, v/v). Subsequently, the eluates were evaporated under a gentle stream of nitrogen (N_2_) at 60 °C. The dry residues were reconstituted in 100 µL of the aqueous mobile phase A (50 mM ammonium formate, pH 3.5) and shaken for 15 min. Twenty µL were injected onto the LC-MS/MS system.

**Method validation**

Six blank serum or whole blood samples from different drug-free pigs were analyzed to check for interfering signals at the multiple reaction monitoring (MRM) transitions of the analytes or the stable-isotope-labeled internal standard (SIL-IS). Two zero samples per matrix containing only SIL-IS were analyzed to check for interference at the retention time of the SIL-IS. In addition, two blank serum or whole blood samples and two zero samples spiked with several opioids were analyzed as described before. The following drugs were tested: U-51754, U-47931E, methoxyacetylfentanyl, fentanyl, norfentanyl, oxycodone, morphine, hydromorphone, hydrocodone, codeine, 6-MAM, methadone, dihydrocodeine, EDDP, tilidine, nortilidine (100 ng/mL each).

Recovery (RE), matrix effects (ME), and process efficiency (PE) were estimated according to Matuszewski et al. (2003) by preparing three different sets of quality control (QC) LOW and HIGH samples. Sample set one represented neat standard solution, sample set two blank matrices spiked after extraction, and sample set three was spiked before extraction. Drug-free serum and blood from different pigs were used to achieve six different sets per matrix. RE was calculated by comparison of the absolute peak areas of sample set three and two. ME was estimated by comparison of the peak areas of set two with those of set one. PE was obtained by comparing peak areas of set three with those of set one.

The linearity of the calibration range was examined by spiking blank serum or whole blood samples with 50 µL of the corresponding calibration standard. Each calibration level was analyzed six times and the peak area ratios between analyte and SIL-IS were plotted against the drug concentration. Linear regression was performed ~~via~~ with the Valistat 2.0 software (Arvecon, Walldorf, Germany) using non-weight, a weighted [1/concentration], and a weighted least-square [1/(concentration)²] regression model and different statistical tests (Grubbs test, F test, Mandel test). The lowest point of the calibration curve determined in serum or whole blood was defined as lower limits of quantification (LLOQ).

Limits of detection (LODs) were estimated by preparing different concentrations in drug-free serum or whole blood. For U-47700, tramadol and their metabolites concentrations of 0.02, 0.05, 0.07, 0.1, 0.12, 0.15, 0.17, 0.2, 0.25, 0.05, 1.0, 1.5, 2.0 2.5, 3.0, 3.5, 4.0, 4.5, and 5.0 ng/mL were prepared in serum. In whole blood, concentrations of 0.1, 0.15, 0.2, 0.25, 0.3, 0.35, 0.4, 0.45, 0.5, 3.5, 4.0, 4.5, 5.0, 5.5, and 6.0 ng/mL were prepared. LODs were determined by signal-to-noise ratios of the quantifier and qualifier MRM transitions of 3:1.

For accuracy and precision tests, 1 mL aliquots of blank pig serum or whole blood were spiked freshly on each of 8 days with 50 µL of QC LOW or HIGH solution. They were analyzed in duplicates and calculated via daily calibration curves. Calculation of bias values, repeatability, and intermediate precision was performed using Valistat 2.0 software.

For determination of the dilution integrity for concentrations higher than the calibration range, blank serum or whole blood samples were spiked with analyte concentrations tenfold the QC HIGH, diluted 1:10 and analyzed in duplicates on 8 days.

In order to estimate the stability of processed samples under conditions of a liquid chromatography-tandem mass spectrometry (LC-MS/MS) analysis session, QC LOW and HIGH samples (n = 6 each) were prepared in blank serum or whole blood and then pooled at each concentration level. They were analyzed at time intervals of 60 min over a total run time of 15 h. Stability was calculated by linear regression with the Valistat 2.0 software by comparison of absolute peak areas.

For determination of freeze/thaw stability, QC LOW and HIGH samples (n = 6 each) were prepared in blank serum or whole blood and analyzed before and after three freeze and thaw cycles. Each freeze/thaw cycle was processed as a 21 h freezing period at - 20 °C followed by a thawing period over 3 h at room temperature. For determination of long-term stability QC LOW and HIGH samples (n = 6 each) were prepared in blank serum or whole blood and analyzed before and after a freezing period of 30 days at - 20 °C.

Carry-over effects were estimated during the analysis of the authentic pig serum and whole blood samples obtained from the toxicokinetic study. Therefore, one blank sample was analyzed between two authentic samples with high concentrations.

**Surgical Procedure**

In accordance with previous studies (Schaefer et al. 2016, 2017, 2018; Nordmeier et al. 2020, 2021), the animals were treated with ketamine hydrochloride (30 mg/kg, Ursotamin; Serumwerk Bernburg, Bernburg, Germany), xylazine hydrochloride (2.5 mg/kg, Rompun; Bayer, Leverkusen, Germany), and 1 mg atropine (Braun, Melsungen, Germany) by intramuscular injection. Anesthesia was maintained with isoflurane (2-4%, Forene, AbbVie, Ludwigshafen, Germany) and mechanically ventilation occurred with a mixture of oxygen and air (1:2 v/v; FiO_2_ of 0.30; Respirator ABV-U; F. Stephan GmbH, Gackenbach, Germany) and a tidal volume of 10-12 mL/kg. A catheter was placed in the left ear vein for fluid (sodium chloride 0.9% [8 mL/kg/h], Braun, Melsungen, Germany) replacement. Sample collection, i.v. drug administration, and monitoring of mean central venous pressure was made sure by the placement of a triple-lumen 7F central venous catheter (Certofix Trio, Braun, Melsungen, Germany) into the jugular vein. For assurance of urine collection, a suprapubic catheter (Cystofix, Braun, Melsungen, Germany) was placed into the bladder. Prior to the drug administration, the pigs were allowed to stabilize for 10-15 min.

**LC-MS/MS conditions**

As already described in a previous study (Nordmeier et al. 2021), an AB SCIEX (Darmstadt, Germany) API 3200 QTrap-MS/MS was coupled to a Shimadzu Prominance HPLC equipped with two solvent delivery units (LC-20AD), a communication bus module (CBM-20A), an autosampler (SIL-20AC), a degasser (DGU-20), and a column oven (CTO-10AC) for the analyzation of serum and whole blood samples. Positive ionization was reached using electrospray ionization (ESI).

Chromatographic separation was performed with a Waters (Wexford, Ireland) Sunfire C_18_ (150 x 2.1 mm, 3.5 µm) analytical column and gradient elution using mobile phase A and B (0.1% formic acid in acetonitrile). The total runtime was about 16 min. The gradient was as follows: starting with 25% eluent B, min 1.5 to 10 ramping to 36% eluent B, min 10 to 11 ramping to 95% eluent B, holding 95% eluent B for 1 min, min 12 to 12.5 reducing to 25% eluent B, min 12.5 to 16 holding 25% eluent B. The flow rate was adjusted at 0.3 mL/min, the injection volume at 20 µL, and the oven temperature at 30 °C.

In positive ESI mode, multiple-reaction monitoring (MRM) was used with a dwell time of 100 ms. Detection of the compounds was applied with three transitions per precursor ion for U-47700 and *N*-desmethyl-U-47700, and two for tramadol and ODT, respectively. Method parameters are provided in Table S1. The source and gas parameters were set as follows: curtain gas (N_2_) – 35 psi, collision gas (N_2_) – medium, temperature – 500 °C, ion source gas 1 (N_2_) – 50 psi, ion source gas 2 (N_2_) – 80 psi, ion spray voltage – 5500 V. Data acquisition was performed with Analyst software Version 1.6.

**S2 Results**

**Method validation**

The analysis of blank pig serum, whole blood, or zero samples revealed no interferences. There were also no interferences in analyzed samples spiked with several opioids.

The full ME, RE, and PE data are shown in Table S2. In general, the matrix effects were in the acceptable range of ± 25% according to the guidelines of the GTFCh (Peters et al. 2009) except for tramadol and ODT in serum QC LOW and whole blood QC LOW and HIGH samples.

LOD values and the calibration ranges are shown in Table S3 and S4. For the calibration, a weighted (1/x²) quadratic regression model was applied for all analytes in both matrices.

The accuracy bias and the relative standard deviation (RSD) values from intra- and interday precision tests are listed in Table S2 and S3. Bias values were within the acceptable interval of ± 15%. The RSD values ≤ 15% are acceptable (Peters et al. 2009). RSD values for all analytes were in this range as well. Sample dilution did not affect these criteria.

All analytes were stable for at least 12 h at 15 °C. A decrease in the peak area > 25% during the tested period indicates instability of the analyte (Peters et al. 2009). During three freeze/thaw cycles, no degradation of any analyte was observed as well as after a freezing period of 30 days at - 20 °C. Mean values of stability samples should be in a range of 90-100% of the control samples (Peters et al. 2009).

During analysis, no carry-over effects were observed.

**S3 Discussion**

**Validation and applicability of the method**

A novel LC-MS/MS method for simultaneous determination of U-47700, tramadol, and their main metabolites was developed in serum and whole blood. All criteria for a successful validation according to the guidelines of the GTFCh (Peters et al. 2009) were fulfilled with minor exceptions in terms of ME and RE. In general, ME was in the acceptable range, but especially in the whole blood samples, signals were suppressed by the matrix. In the case of tramadol and ODT, a markable suppression of the signals was determined in whole blood samples, whereas in serum samples suppression was not that pronounced. One reason for that could be the difference in the composition of whole blood and serum. Whole blood contains more solid components, which aggravate SPE and maybe affect analyte extraction as well. However, in the context that ME data were carried out without the usage of SIL-IS, ME probably will be compensated in routine analysis using SIL-IS and for both compounds, tramadol and ODT, SIL-IS was used. Furthermore, the low standard deviation (SD) indicates a good reproducibility of the method and its results. Nevertheless, ME in serum were acceptable and TK data were mainly collected in serum. The higher SD observed for ME of tramadol and ODT in whole blood indicated matrix-dependent differentially pronounced ME or RE. According to the guidelines of the GTFCh (Peters et al. 2009), these values were determined without SIL-IS and they were not separately tested for the SIL-IS. But some preliminary experiments before the validation revealed that the SIL-IS and the compounds showed a similar behavior concerning ME and RE. Furthermore, ME and RE values were comparable within the same matrix for the analyte and the SIL-IS. This fact indicates that the usage of the SIL-IS in the quantification will compensate ME and RE effects of the analytes and the influence of different matrices on the quantification could be neglected. Another conspicuousness was the low RE in terms of U-47700 and its metabolite in the whole blood QC HIGH samples. Values of RE > 50% are permitted by the GTFCh (Peters et al. 2009) and in our method only values of about 35% were reached in whole blood samples, whereas values for serum samples were about twice as high und thus in the acceptable range. PE values were low, too. Hence, a signal suppression by whole blood alone might not be the reason for the low RE. As described above in terms of ME, also in this case the combination of whole blood and SPE might be responsible for the low RE of this substance. However, SD values were very low for this RE values, indicating results of a very good reproducibility. Furthermore, all signals of the whole blood samples showed acceptable intensities, low concentrations were less influenced by this effect and, thus, a quantification of low concentrations in whole blood could be guaranteed. In addition, as for ME, these data were carried out without the usage of an~~d~~ SIL-IS and, thus, RE might be compensated in routine analysis using SIL-IS as well.

Regarding U-47700 and its metabolite, LOD and LLOQ values obtained for serum in this study were in the same range as those determined in the study by Smith et al. (2019) with the exception of some minor differences. LOD and LLOQ values were slightly higher in our study. LOD values for whole blood for both substances were in agreement with those from the method developed by Seither et al. (2017), whereas LLOQ values were lower in our method compared to that of Gerace et al. (2018), who applied a fast protein precipitation approach. In terms of tramadol, LOD and LLOQ values were slightly lower in our study compared to that of Fernández et al. (2013), who established a quantification method for 26 opioids in whole blood after SPE. LLOQ values in serum were in the same range for tramadol and ODT, as determined in the study of Meyer et al. (2015).

The validated method was applied to a TK study following i.v. administration of U-47700 or tramadol to 6 pigs, respectively. The results obtained in one group of the pigs receiving the same drug were in good agreement with each other and the method was suitable to determine the serum and whole blood concentration-time profiles of both substances ~~as well as~~ and their metabolites. Regarding U-47700, only a few samples had to be diluted due to concentrations above the calibration range. Eight hours following administration, most whole blood samples displayed higher concentrations than the LOD, whereas some concentrations were lower than the LLOQ, but the analytes were still detectable. Regarding the serum samples, all determined concentrations were higher than the LLOQ. In terms of tramadol, many samples had to be diluted due to much higher concentrations above the calibration range. Caused by the higher dosage, higher serum/whole blood concentrations were estimated, but also low concentrations at the end of the experiment because of a great concentration decrease in the organism during the experiment. However, in terms of the linearity of the calibration curve, a broader calibration range was not possible. Due to the medium sensitivity of tramadol and ODT within this applied method and the used MS/MS, many samples showed concentrations lower than the LOD/LLOQ at the end of the experiment in terms of whole blood samples, but both substances were still detectable in each sample. In terms of serum samples, most of the determined concentrations were higher than the LLOQ.

Usually, plasma is the matrix of choice in PK/TK studies. Due to the high popularity of serum and whole blood and the minor relevance of plasma in forensic toxicology, the authors examined this TK study in serum and whole blood and validated the LC-MS/MS method in these matrices. Nevertheless, due to only minor differences between the matrices of serum, plasma and whole blood, the validated method might also applicable for the quantification of U-47700, tramadol and their metabolites in plasma.

**S4 popTK modeling: differential equations and parameter calculation**

A three-compartment linear mammillary model was implemented in NONMEM^®^ using ADVAN11 TRANS4 subroutine as described below:

**1** = CENTRAL: DADT(1) = – CL/V1*A(1) + Q2/V2*A(2) –Q2/V1*A(1) + Q3/V3*A(3) – Q3/V1*A(1)

**2** = PERIPHERAL 1: DADT(2) = Q2/V1*A(1) – Q2/V2*A(2)

**3** = PERIPHERAL 2: DADT(3) = Q3/V1*A(1) – Q3/V3*A(3)

**4** = OUTPUT

**NONMEM model file** and **parameters calculation** used in the single species scaling from pig to human for tramadol using LBF on CL and WGT on all patameters:

**$PROBLEM** PK

**$INPUT** ID TIME AMT RATE CMT DV MDV WGT AMTKG SPECIES BRAIN BrainWGT WGT1 BILE UDPGT ENQ BRAINTOWGT DOSE DUR WGT2 MLP GFR CF

**$DATA** Human_all.csv IGNORE=@

**$SUBROUTINES** ADVAN11 TRANS4

**$PK**

TVCL = THETA(1)*((WGT/42.83)**THETA(7))*((364/73)/(517.3/25.5))

CL = TVCL * EXP(ETA(1))

TVV1 = THETA(2)*((WGT/42.83)**THETA(8))

V1 = TVV1 * EXP(ETA(2))

TVQ2 = THETA(3)*((WGT/42.83)**THETA(9))

Q2 = TVQ2 * EXP(ETA(3))

TVV2 = THETA(4)*((WGT/42.83)**THETA(10))

V2 = TVV2 * EXP(ETA(4))

TVQ3 = THETA(5)*((WGT/42.83)**THETA(11))

Q3 = TVQ3 * EXP(ETA(5))

TVV3 = THETA(6)*((WGT/42.83)**THETA(12))

V3 = TVV3 * EXP(ETA(6))

S1 = V1

**$ERROR**

IPRED=F

DEL=0

IF (IPRED.EQ.0) DEL=0.0001

W=F

IRES=DV-IPRED

IWRES=IRES/(W+DEL)

Y=IPRED+W*EPS(1)

**$THETA**

(0, 78.9) FIX ; CL (L/h)

(0, 53.6) FIX ; V1 (L)

(0, 30.8) FIX ; Q2 (L/h)

(0, 74.7) FIX ; V2 (L)

(0, 147) FIX ; Q3 (L/h)

(0, 28.4) FIX ; V3 (L)

(1) FIX ; WGT on CL

(1) FIX ; WGT on V1

(1) FIX ; WGT on Q2

(1) FIX ; WGT on V2

(1) FIX ; WGT on Q3

(1) FIX ; WGT on V3

**$OMEGA**

0.0394 FIX ; IIV CL

0.0745 FIX ; IIV V1

0.133 FIX ; IIV Q2

0.102 FIX ; IIV V2

0 FIX ; IIV Q3

0 FIX ; IIV V3

**$SIGMA**

0.0226 ; RV Proportional error

**$EST** METHOD=1 INTER MAXEVAL=9999 NOABORT PRINT=1 POSTHOC

**$COV**

**$TABLE** ID TIME IPRED IWRES CWRES FILE=sdtab3105_015 ONEHEADER NOPRINT

**$TABLE** CL V1 V2 V3 Q2 Q3 ONEHEADER NOPRINT FIRSTONLY FILE=patab3105_015

| **Table S1** MRM transitions and MS conditions for all analytes and the stable-isotope-labeled internal standard with collision energy (CE), declustering potential (DP), entrance Potential (EP), and collision cell exit potential (CXP). | | | | | | | |
| --- | --- | --- | --- | --- | --- | --- | --- |
| Analyte | RT (min) | Precursor Ion  (Q1, m/z) | Product Ion  (Q3, m/z) | DP (V) | EP (V) | CE (V) | CXP (V) |
| U-47700 | 7.99 | 329.0 | 173.0 | 41 | 6.5 | 39 | 6 |
|  |  | 329.0 | 284.2 | 41 | 6.5 | 19 | 16 |
|  |  | 329.0 | 204.1 | 41 | 6.5 | 33 | 6 |
| *N*-desmethyl-U-47700 | 7.42 | 314.9 | 284.0 | 41 | 7 | 21 | 12 |
|  |  | 314.9 | 173.1 | 41 | 7 | 35 | 6 |
|  |  | 314.9 | 204.1 | 41 | 7 | 35 | 8 |
| Tramadol | 2.88 | 264.3 | 58.2 | 21 | 6.5 | 37 | 4 |
|  |  | 264.3 | 57.4 | 21 | 6.5 | 37 | 58 |
| *O*-desmethyltramadol | 2.33 | 250.2 | 57.9 | 21 | 10 | 39 | 8 |
|  |  | 250.2 | 57.1 | 21 | 10 | 77 | 4 |
| *N*-desmethyl-U-47700-*d*_3_ | 7.36 | 318.1 | 287.2 | 41 | 9 | 21 | 10 |
|  |  | 318.1 | 176.1 | 41 | 9 | 39 | 6 |
|  |  | 318.1 | 148.1 | 41 | 9 | 65 | 4 |
| Tramadol-*C*_13_-*d*_3_ | 2.84 | 268.2 | 58.0 | 31 | 4 | 37 | 8 |
|  |  | 268.2 | 57.1 | 31 | 4 | 89 | 56 |
| *O*-desmethyltramadol-*d*_6_ | 2.32 | 256.2 | 64.1 | 36 | 3 | 29 | 4 |
|  |  | 256.2 | 77.0 | 36 | 3 | 79 | 4 |

| **Table S2** Drug, matrix effects (ME), recovery (RE), and process efficiency (PE) including their relative standard deviation (RSD) for pig serum/whole blood QC LOW and QC HIGH samples. | | | | | | | |
| --- | --- | --- | --- | --- | --- | --- | --- |
| Analyte | Serum | | |  | Whole blood | | |
|  | ME% (RSD%) | RE% (RSD%) | PE% (RSD%) |  | ME% (RSD%) | RE% (RSD%) | PE% (RSD%) |
| OC LOW |  |  |  |  |  |  |  |
| U-47700 | 77.1 (5.6) | 74.9 (7.3) | 57.7 (4.8) |  | 101.4 (11.3) | 70.2 (15.4) | 71.1 (16.5) |
| *N*-desmethyl-U-47700 | 78.0 (5.5) | 72.5 (5.5) | 56.5 (11.6) |  | 104.1 (10.6) | 68.4 (17.4) | 71.2 (16.9) |
| Tramadol | 71.8 (8.9) | 81.0 (4.0) | 58.1 (7.9) |  | 67.2 (28.1) | 76.1 (17.2) | 51.2 (15.6) |
| *O*-desmethyltramadol | 63.7 (8.2) | 89.8 (6.4) | 57.2 (9.3) |  | 36.1 (17.5) | 77.6 (10.0) | 28.0 (9.5) |
| QC HIGH |  |  |  |  |  |  |  |
| U-47700 | 92.7 (2.7) | 67.0 (6.0) | 62.1 (5.3) |  | 110.5 (14.5) | 33.2 (2.0) | 36.6 (6.8) |
| *N*-desmethyl-U-47700 | 91.8 (4.7) | 66.1 (6.0) | 60.7 (6.2) |  | 115.4 (15.0) | 36.3 (4.2) | 42.0 (9.08) |
| Tramadol | 83.9 (6.6) | 80.5 (4.4) | 67.5 (4.9) |  | 76.1 (20.8) | 78.2 (26.4) | 59.5 (14.0) |
| *O*-desmethyltramadol | 77.3 (7.5) | 86.5 (3.7) | 66.8 (6.3) |  | 48.7 (17.7) | 85.6 (37.4) | 41.7 (10.7) |

| **Table S3** Drug, used stable-isotope-labeled internal standard (SIL-IS), limit of detection (LOD), calibration range with lower limits of quantification (LLOQ) as lowest point, nominal concentration, accuracy bias, and the relative standard deviation (RSD) values for repeatability and intermediate precision for pig serum CQ LOW and QC HIGH samples. | | | | | | | | | | | | |
| --- | --- | --- | --- | --- | --- | --- | --- | --- | --- | --- | --- | --- |
| Analyte | SIL-IS | LOD [ng/mL] | Calibration range [ng/mL] | QC LOW | | | |  | QC HIGH | | | |
|  |  |  |  | Nominal conc. [ng/mL] | Accuracy bias [%] | Repeatability RSD [%] | Intermediate Precision RSD [%] |  | Nominal conc. [ng/mL] | Accuracy bias [%] | Repeatability RSD [%] | Intermediate Precision RSD [%] |
| U-47700 | *N*-desmethyl-U-47700-*d*_3_ | 0.05 | 0.25-50 | 0.75 | 1.0 | 9.6 | 9.6 |  | 35 | -0.04 | 7.4 | 11.1 |
| *N*-desmethyl-U-47700 | *N*-desmethyl-U-47700-*d*_3_ | 0.1 | 0.25-50 | 0.75 | 9.3 | 7.0 | 7.0 |  | 35 | -0.3 | 8.6 | 8.6 |
| Tramadol | Tramadol-*C*_13_- *d*_3_ | 0.05 | 0.5-100 | 1.5 | 8.0 | 6.2 | 8.5 |  | 75 | 9.6 | 3.9 | 3.9 |
| *O*-desmethyl-tramadol | *O*-desmethyl-tramadol- *d*_6_ | 1.5 | 5-100 | 7.5 | 8.7 | 7.6 | 7.6 |  | 75 | 2.2 | 7.0 | 10.1 |

| **Table S4** Drug, used stable-isotope-labeled internal standard (SIL-IS), limit of detection (LOD), calibration range with lower limits of quantification (LLOQ) as lowest point, nominal concentration, accuracy bias, and the relative standard deviation (RSD) values repeatability and intermediate precision for pig whole blood CQ LOW and QC HIGH samples. | | | | | | | | | | | | |
| --- | --- | --- | --- | --- | --- | --- | --- | --- | --- | --- | --- | --- |
| Anaylte | SIL-IS | LOD [ng/mL] | Calibration range [ng/mL] | QC LOW | | | |  | QC HIGH | | | |
|  |  |  |  | Nominal conc. [ng/mL] | Accuracy bias [%] | Repeatability RSD [%] | Intermediate Precision RSD [%] |  | Nominal conc. [ng/mL] | Accuracy bias [%] | Repeatability RSD [%] | Intermediate Precision RSD [%] |
| U-47700 | *N*-desmethyl-U-47700-*d*_3_ | 0.1 | 0.5-50 | 2 | 11.28 | 4.02 | 4.94 |  | 35 | 4.89 | 3.46 | 6.94 |
| *N*-desmethyl-U-47700 | *N*-desmethyl-U-47700-*d*_3_ | 0.1 | 0.5-50 | 2 | 4.84 | 3.45 | 4.62 |  | 35 | -1.19 | 4.03 | 5.75 |
| Tramadol | Tramadol-*C*_13_- *d*_3_ | 0.1 | 1-100 | 8 | 4.13 | 3.77 | 6.23 |  | 80 | 3.30 | 2.05 | 3.36 |
| *O*-desmethyl-tramadol | *O*-desmethyl-tramadol- *d*_6_ | 3.5 | 10-100 | 20 | 4.46 | 2.68 | 4.96 |  | 80 | 1.81 | 3.36 | 6.12 |

**Table S5** Overview of the digitized tramadol literature data, used in the prediction of human tramadol concentration-time profiles and interspecies scaling analysis. NA not available, i.v. intravenous

| **ID** | **Species** | **Sex** | **n** | **Weight** | **Administration** | **Dose** | **Matrix** | **Sampling times** | **Reference** |
| --- | --- | --- | --- | --- | --- | --- | --- | --- | --- |
| **Human studies** | | | | | | | | | |
| **1** | Volunteers (immediately after operation) | NA | 6 | 71.2 ± 2.5 kg | single i.v. infusion (over 2 min) | 100 mg | measured in plasma | 0, 0.1, 0.25, 0.5, 0.75, 1, 2, 4, 6, 8, 12, 16, 20, and 24 h after drug administration. | (Yılmaz and Erdem 2015) |
| **2** | Healthy volunteers | male | 12 | 50 to 80 kg  (65 kg mean value) | single i.v. infusion  (over 10 min) | 100 mg  (racemic tramadol) | R(+) and S(-) tramadol enantiomers measured in plasma (forearm vein) | 0, 0.25, 0.5, 0.75, 1, 2, 4, 6, 8, 12 and 24 h after the end of the infusion | (Quetglas et al. 2007) |
| **3** | Healthy volunteers | NA | 12 | NA  (assumed 70 kg) | single i.v. infusion  (over 10 min) | 100 mg  (racemic tramadol) | R(+) and S(-) tramadol enantiomers measured in plasma | 0, 10, 25, 40, and 55 min; and 1, 2, 4, 6, 8, 12, and 24 h after start of infusion. | (Campanero et al. 1999) |
| **4** | Healthy volunteers | NA | 17 | NA  (assumed 70 kg) | multiple i.v. infusion  (assumed bolus) | 25 mg at 0h, followed by 25 mg at 2h, 25 mg at 4h, and 25 mg every 4 hours thereafter through 44h | measured in plasma | 0, 0.15,0.25, 0.5, 0.75, 1, 1.5, 2, 2.25, 2.5, 4, 4.25, 4.5, 5, 6, 8, 12, 16, 20, 24, 32, 40, 44, 44.3, 44.5, 44.8, 45, 45.5, 46, 47, 48 h | (United States Patent 2018) |
| **5** | Healthy volunteers | NA | 17 | NA  (assumed 70 kg) | multiple i.v. infusion (assumed bolus) | 50 mg at 0h, followed by 50 mg at 2h, 50 mg at 4h, and 50 mg every 4 hours thereafter through 44h | measured in plasma | 0, 0.15,0.25, 0.5, 0.75, 1, 1.5, 2, 2.25, 2.5, 4, 4.25, 4.5, 5, 6, 8, 12, 16, 20, 24, 32, 40, 44, 44.3, 44.5, 44.8, 45, 45.5, 46, 47, 48 h | (United States Patent 2018) |
| **Animal studies** | | | | | | | | | |
| **6** | C57Bl/6 Mice | 3 male/ 3 female | 6 | 20 - 30 g  (25 g mean value) | i.v. bolus | 25 mg/kg | measured in plasma (from tail-vein) | composite PK profile: 2 samples per mouse: 5, 15, 30 min and 1, 2, 4 hours post i.v. | (Evangelista Vaz et al. 2018) |
| **7** | Sprague-Dawley rats | male | 6 | 250 - 300 g  (275 g mean value) | single i.v. bolus | 10 mg/kg | measured in plasma (from jugular vein) | tramadol 18 h after pretreatment with either MDMA or normal saline, blood samples at appropriate time intervals (0–300 min) | (Jamali et al. 2017) |
| **8** | Rhesus Macaques (Macaca Mulatta) | male | 4 | 17 ± 2 kg | single i.v. bolus | 1.50 mg/kg | measured in serum (from cephalic vein) | prior and 2, 5, 10, 15, 23, 45 min and 1, 1.5, 2, 3, 4, 6, 8, 10 h after drug administration | (Kelly et al. 2015) |
| **9** | Mixed breed dogs | male | 6 | 22 to 32 kg (28.8 kg mean value) | single i.v. bolus | 1.0 mg/kg | measured in plasma (from jugular vein) | 0, 1, 2, 5, 10, 20, 40, and 60 min and at 2, 4, 6, 12, and 24 h. | (McMillan et al. 2008) |
| **10** | Adult llamas | male | 6 | 107 to 140 kg (123.5 kg mean value) | single i.v. bolus (over 1 min.) | 2.0 mg/kg | measured in plasma (from jugular vein) | 0, 1, 3, 5, 10, 15, 20, 30, 45 min and 1, 1.5, 2, 4, 8, 12, 18, 24 and 48 h following administration | (Cox et al. 2011) |
| **11** | Healthy adult horses | 4 male / 5 female | 9 | 563.7 ± 49.1 kg | single i.v. bolus | 0.5, 1.5 and 3 mg/kg | measured in plasma (from jugular vein) | at time 0 and at 5, 10, 15, 30 and 45 min and 1, 1.5, 2, 2.5, 3, 3.5, 4, 5, 6, 8, 12, 18, 24, 36, 48 and 72 h post administration | (Knych et al. 2013) |

**A**


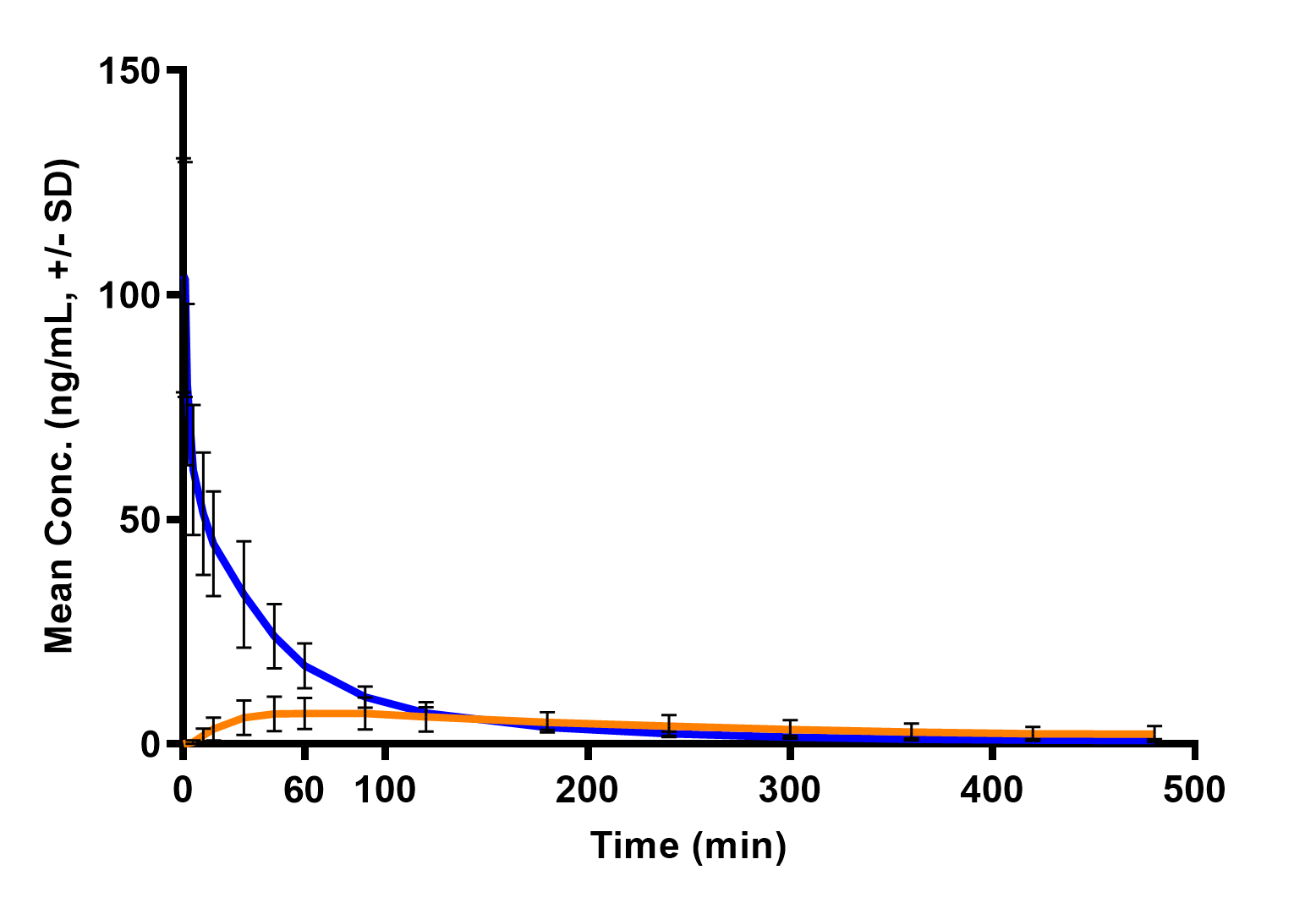


**B**


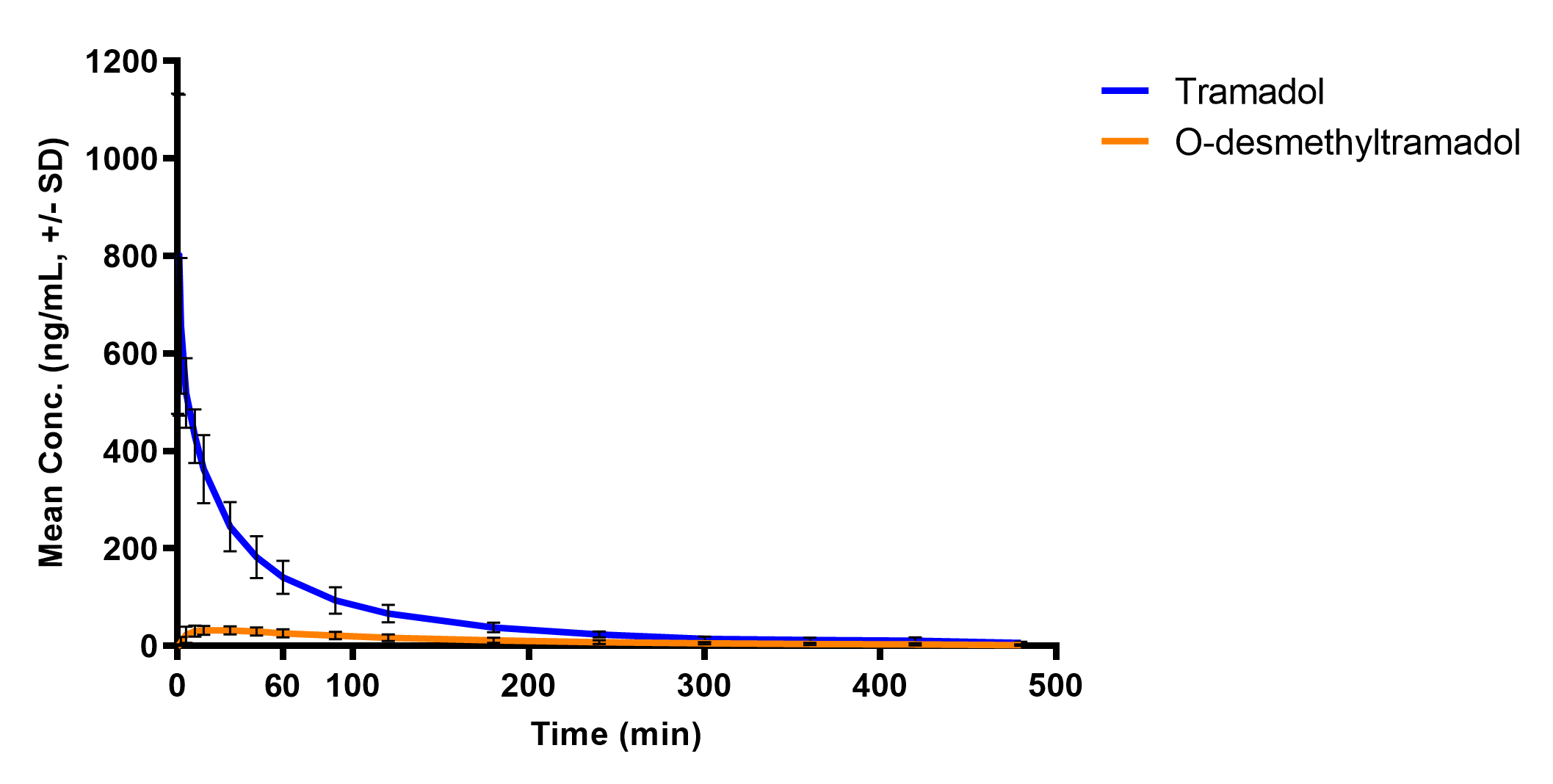


**Fig. S1** Mean concentration-time profiles (n = 6 each) including standard deviation (SD) of **A.** U-47700 (blue line) and *N*-desmethyl-U-47700 (orange line) after single i.v. administration of a100 µg/kg body weight (BW) dose, and **B.** Tramadol (blue line) and *O*-desmethyltramadol (orange line) after single i.v. administration of a 1000 µg/kg BW dose determined in pig serum.

**A**


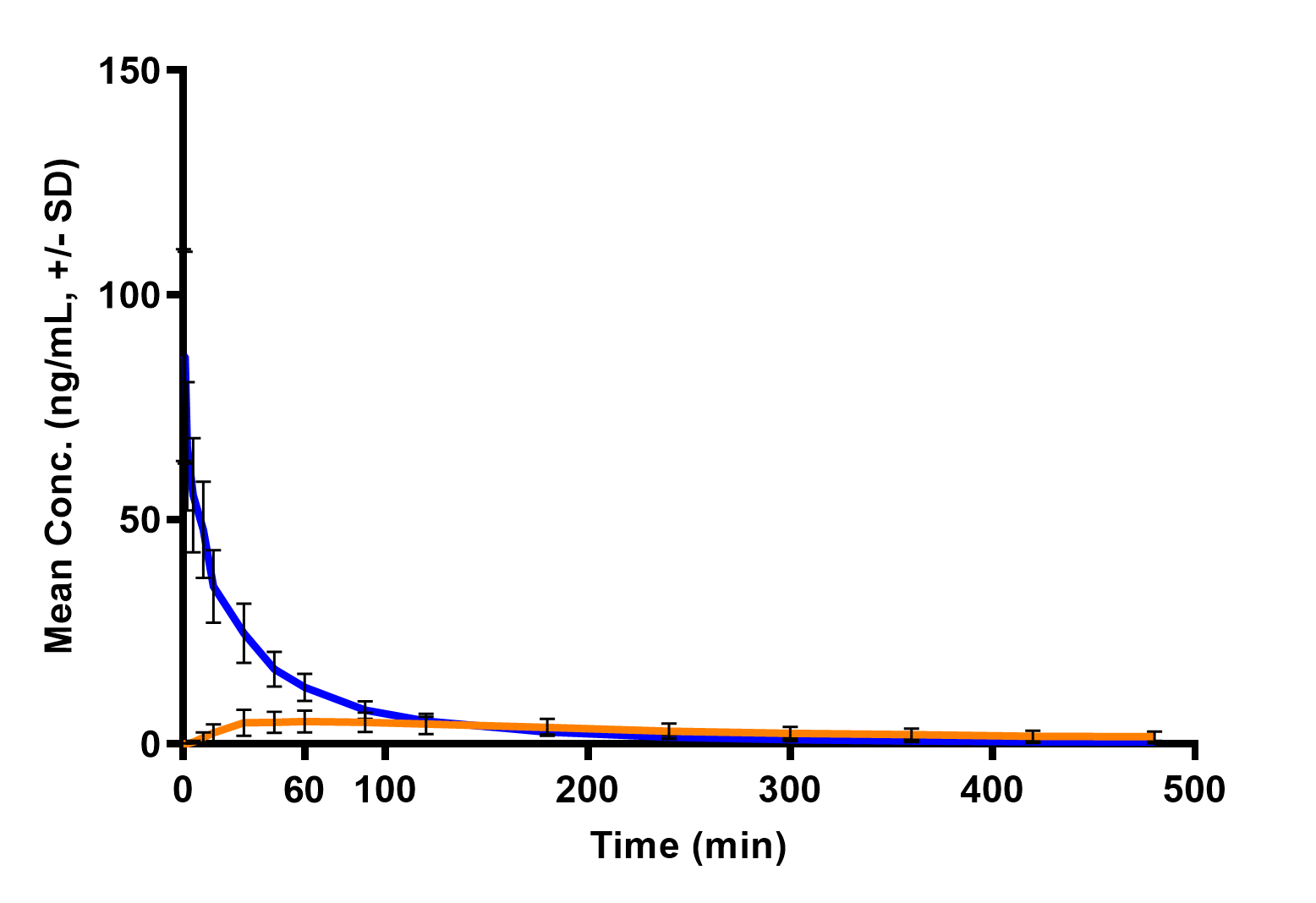


**B**


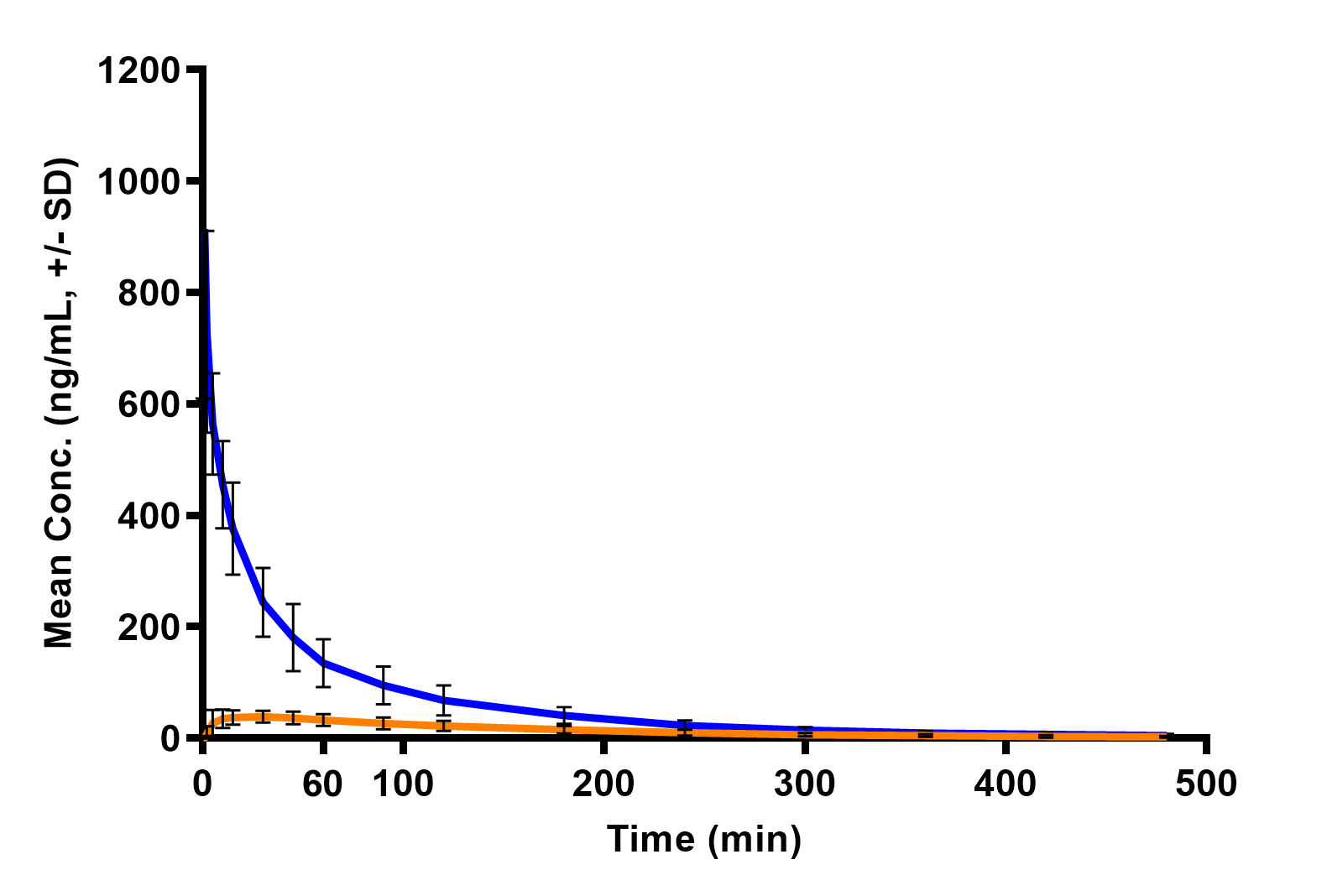


**Fig. S2** Mean concentration-time profiles (n = 6 each) including standard deviation (SD) of **A.** U-47700 (blue line) and *N*-desmethyl-U-47700 (orange line) after single i.v. administration of a 100 µg/kg body weight (BW) dose, and **B.** Tramadol (blue line) and *O*-desmethyltramadol (orange line) after single i.v. administration of a 1000 µg/kg BW dose determined in pig whole blood.

**A**


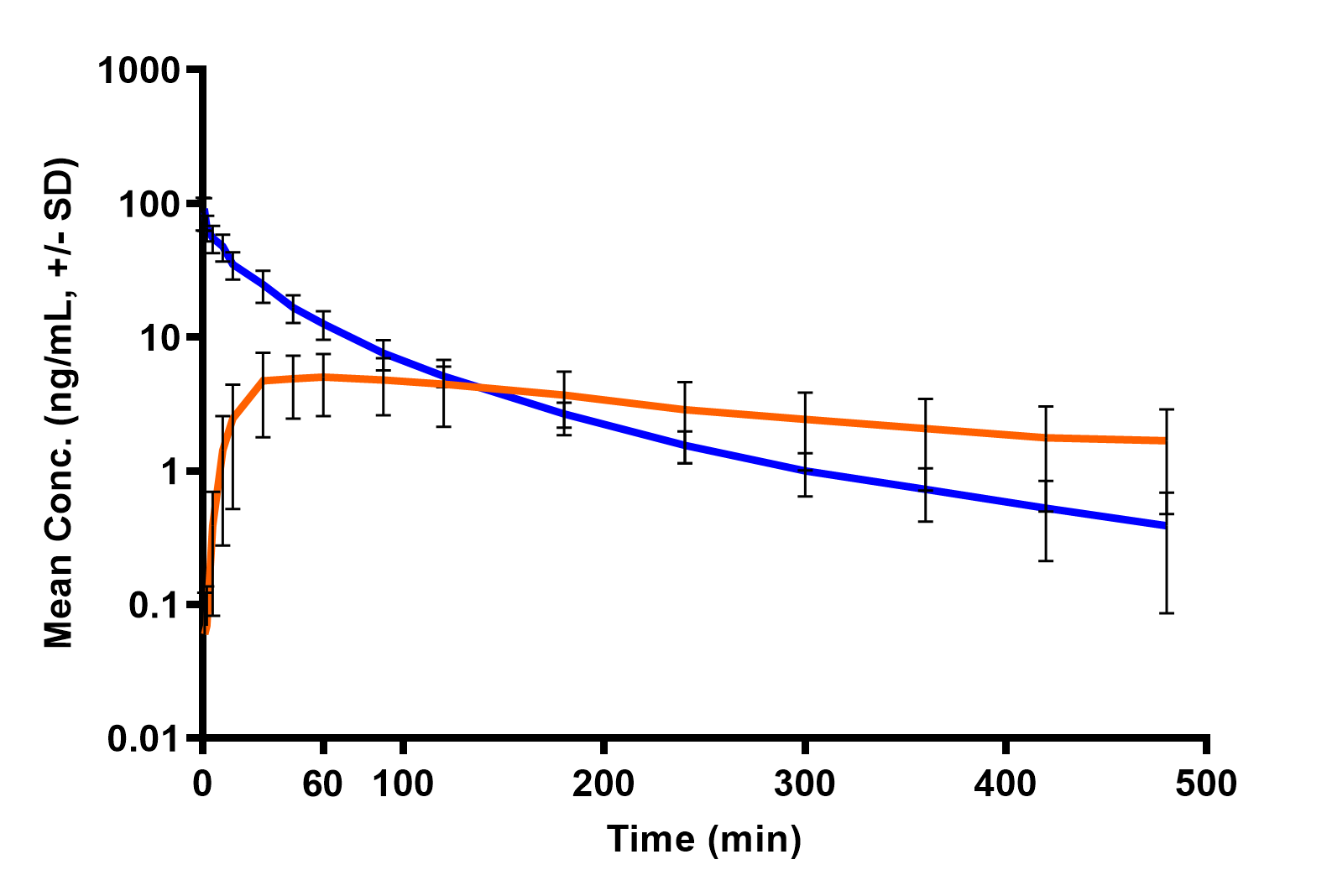


**B**


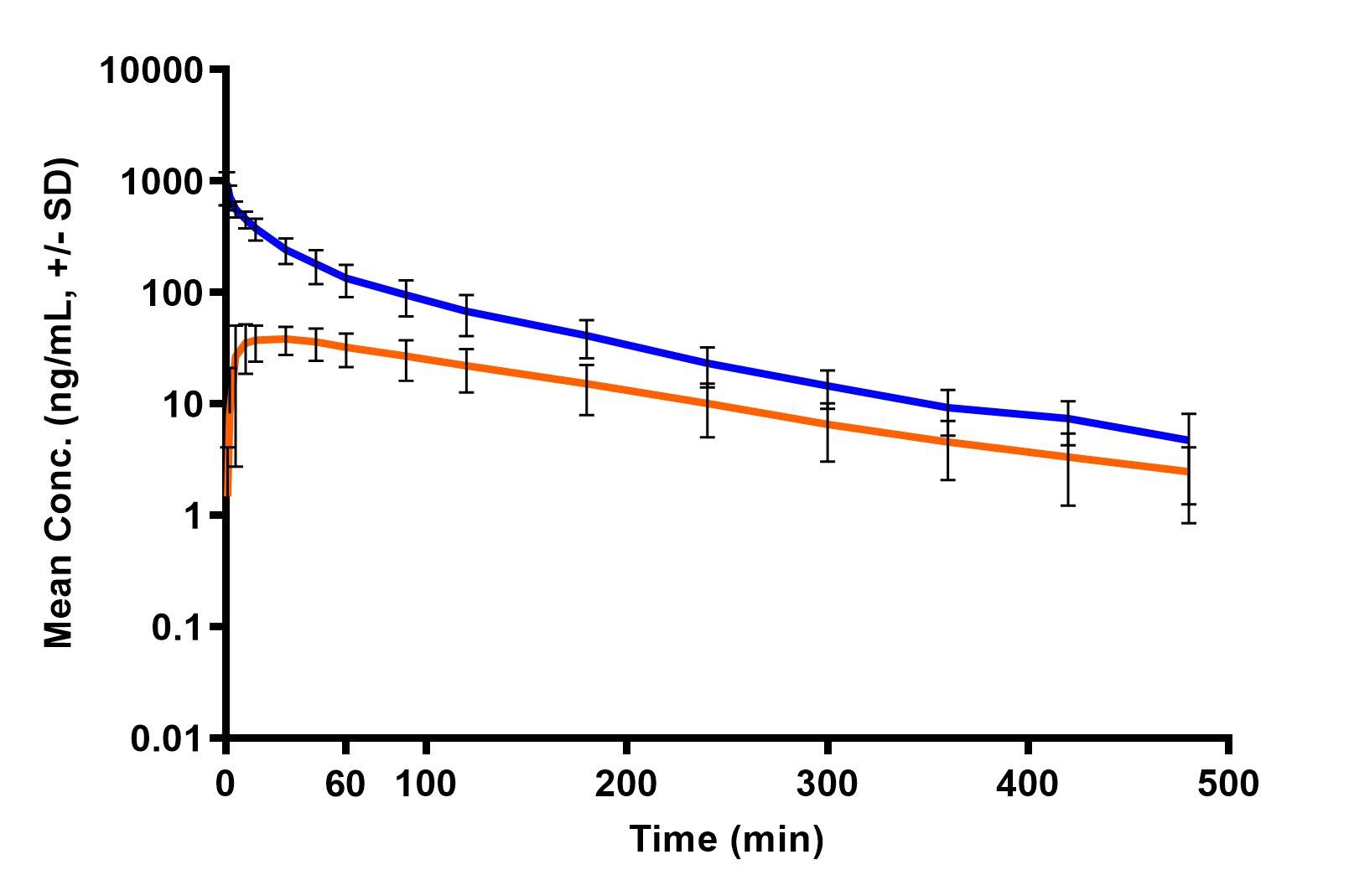


**Fig. S3** Semi-logarithmic plot of the mean concentration-time profiles including standard deviation (SD) of **A.** U-47700 (blue line) and *N*-desmethyl-U-47700 (orange line) after single i.v. administration of a 100 µg/kg body weight (BW) dose, and **B.** Tramadol (blue line) and *O*-desmethyltramadol (orange line) after single i.v. administration of a1000 µg/kg BW dose determined in pig whole blood.


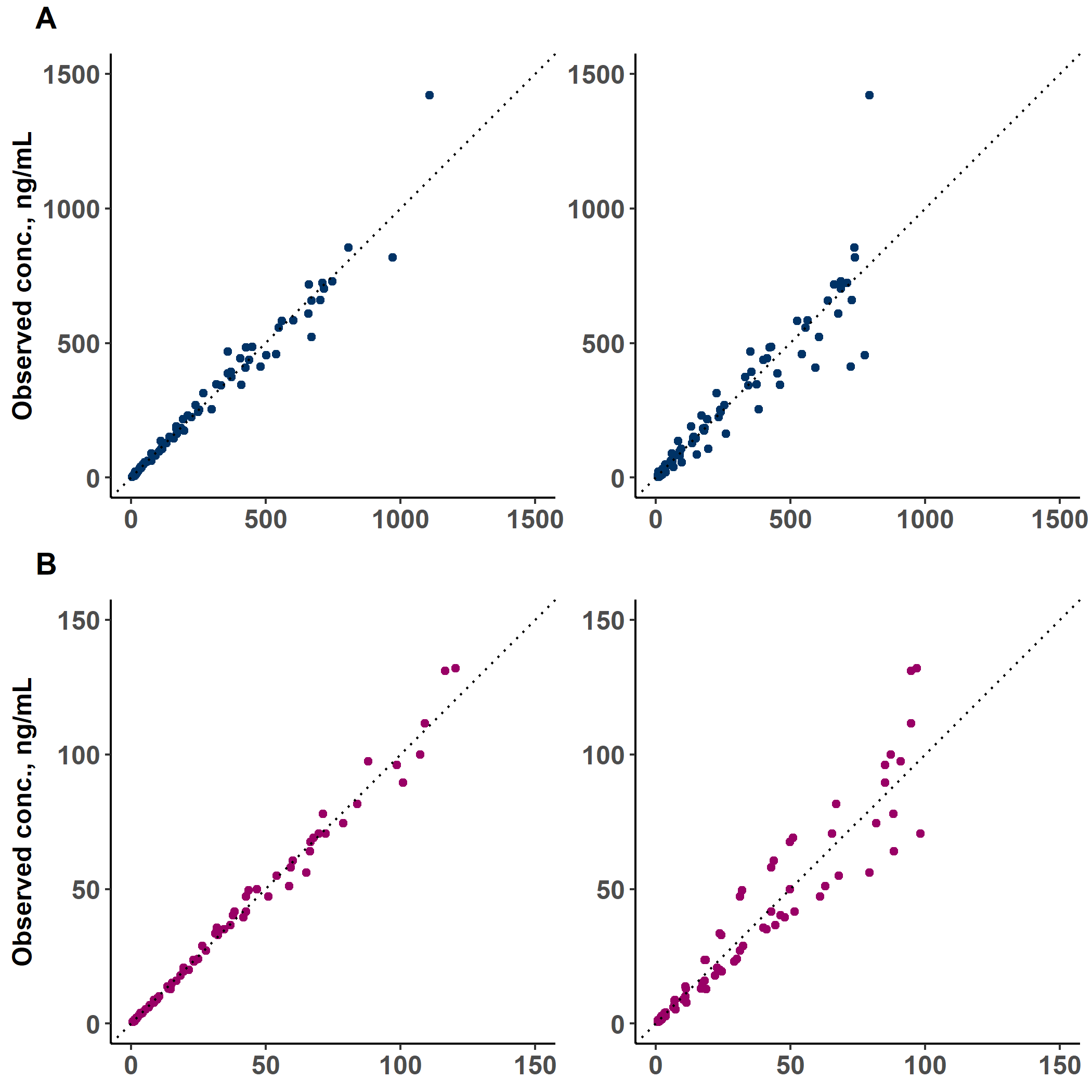


**Fig. S4** Goodness-of-fit plots for the final population (pop) toxicokinetic (TK) model for tramadol (**A**) and U-47700 (**B**). On the left-hand side are plots of observations versus individual predictions. On the right-hand side are plots of observed serum concentrations versus population predictions. The black dotted line represents the line of identity.


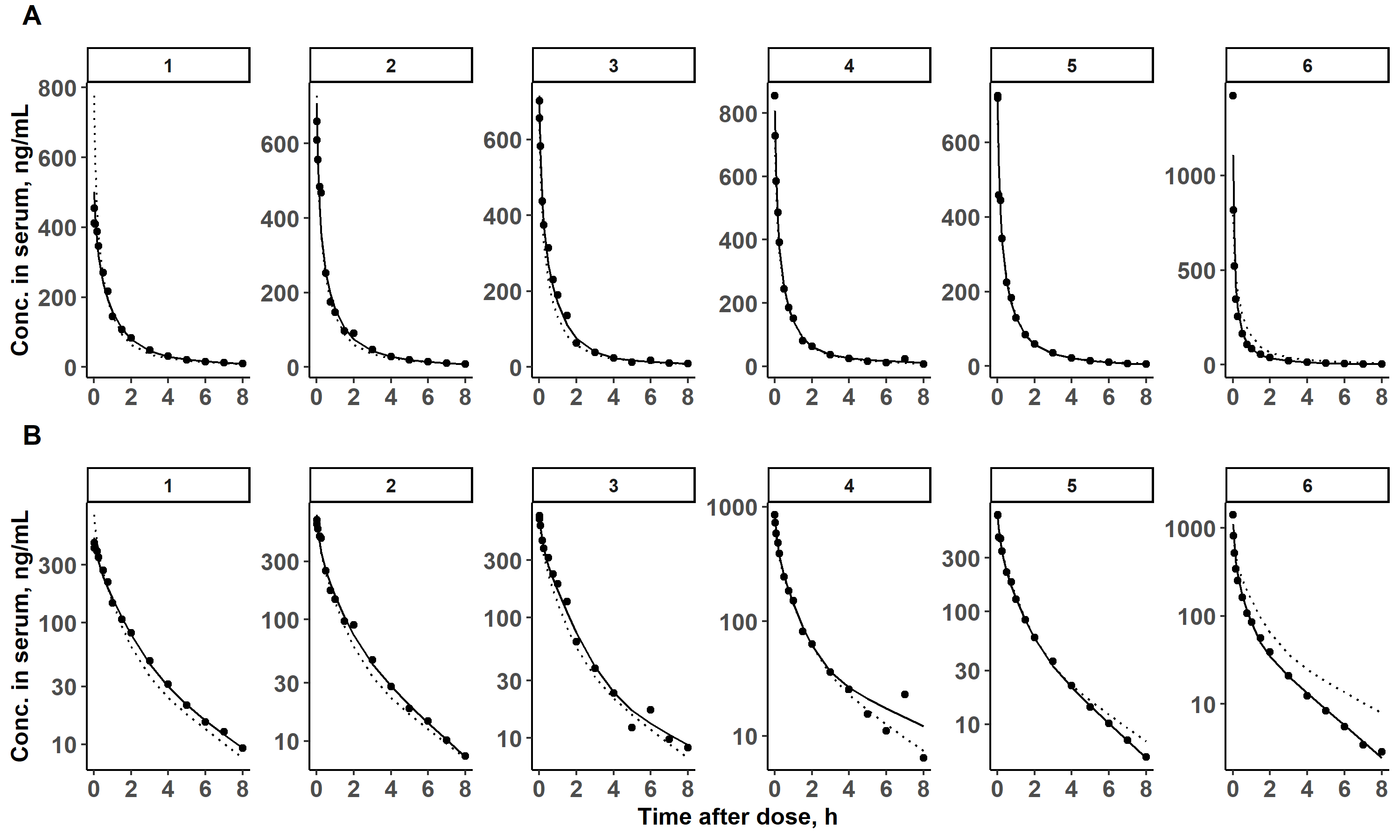


**Fig. S5a** Individual plots for tramadol serum concentration in pigs (n = 6; pig 1-6) on the **A.** linear and **B.** semi-logarithmic scale. The dots represent observed concentrations. Solid lines depict individual predictions (IPRED), dotted lines show population predictions (PRED).


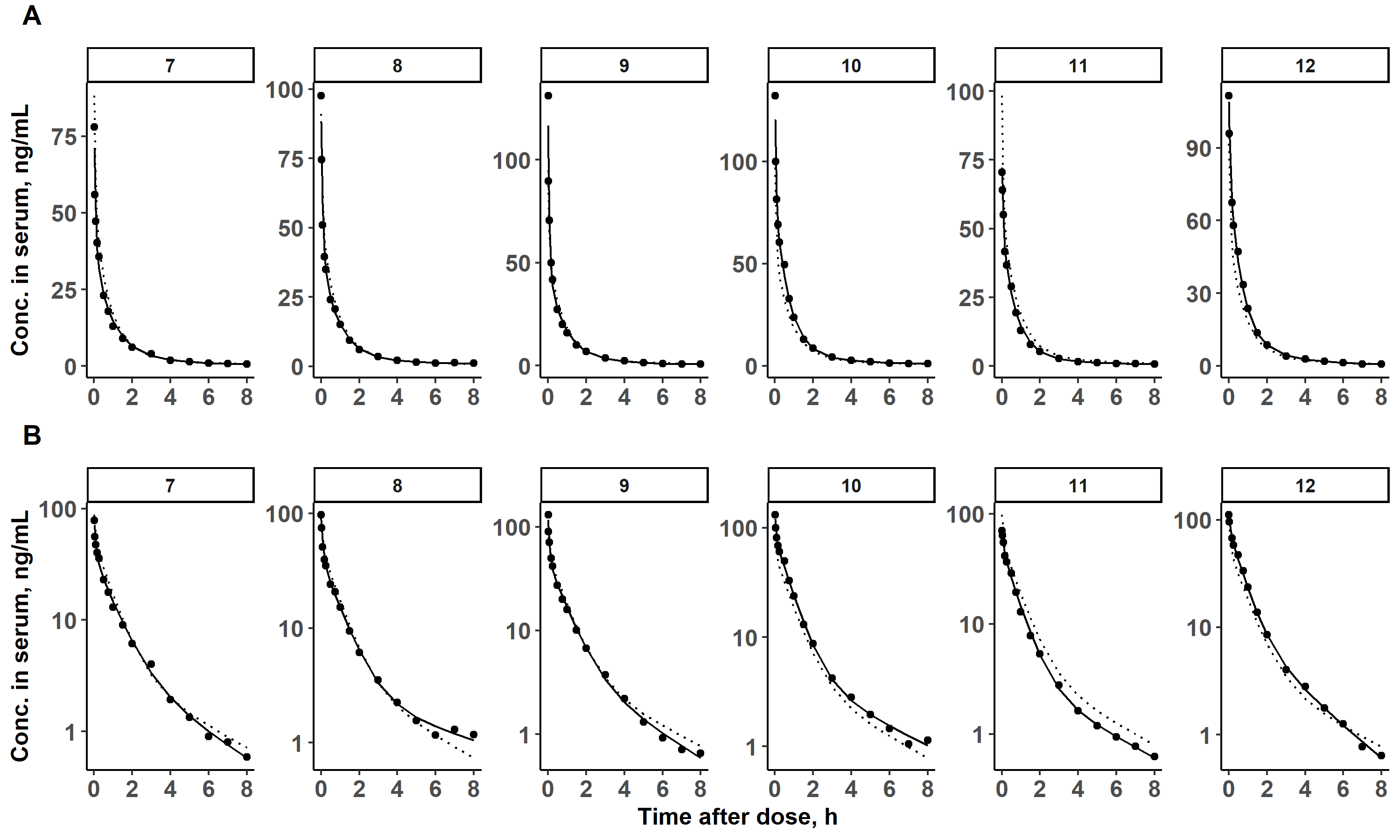


**Fig. S5b** Individual plots for U-47700 serum concentration in pigs (n = 6; pig 7-12) on the **A.** linear and **B.** semi-logarithmic scale. The dots represent observed concentrations. Solid lines depict individual predictions (IPRED), dotted lines show population predictions (PRED).


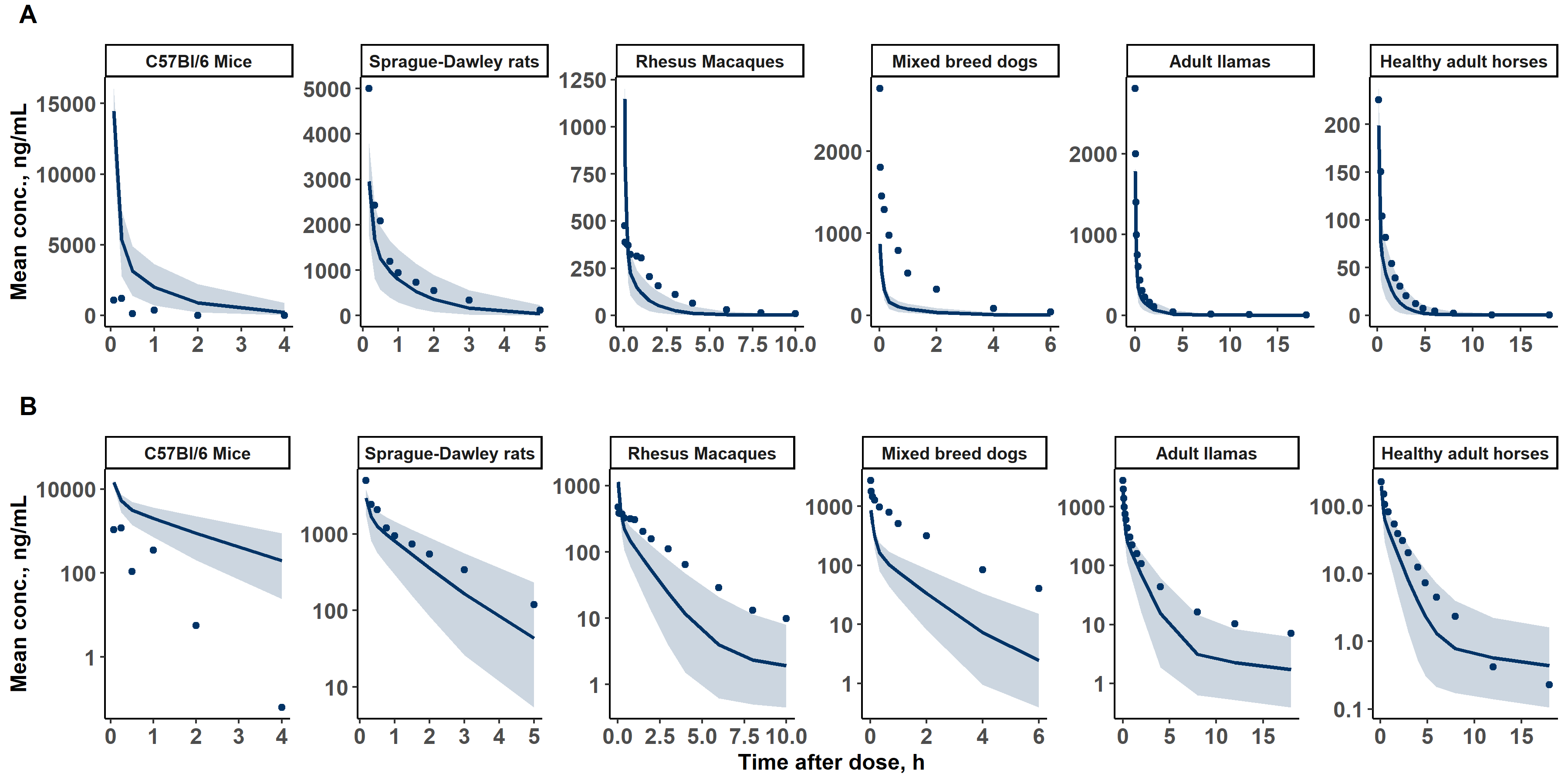


**Fig. S6** Visual predictive checks (VPCs) of three-compartmental popTK model (with IIV on central clearance) for tramadol in different animal species on the **A.** linear and **B.** semi-logarithmic scale. The dots represent observed concentrations. The lines depict the median of the predicted concentrations and the shaded area is the 90% confidence interval of the predictions after 1000 simulations.

**References**

Campanero MA, Calahorra B, Valle M, Troconiz IF, Honorato J (1999) Enantiomeric separation of tramadol and its active metabolite in human plasma by chiral high-performance liquid chromatography: Application to pharmacokinetic studies. Chirality 11:272–279. https://doi.org/https://doi.org/10.1002/(SICI)1520-636X(1999)11:4<272::AID-CHIR3>3.0.CO;2-I

Cox S, Martín-Jiménez T, Amstel S, Doherty T (2011) Pharmacokinetics of intravenous and intramuscular tramadol in llamas. J Vet Pharmacol Ther 34:259–264. https://doi.org/10.1111/j.1365-2885.2010.01219.x

Evangelista Vaz R, Draganov DI, Rapp C, Avenel F, Steiner G, Arras M, Bergadano A (2018) Preliminary pharmacokinetics of tramadol hydrochloride after administration via different routes in male and female B6 mice. Vet Anaesth Analg 45:111-112. https://doi.org/10.1016/j.vaa.2016.09.007

Fernandez M, Wille SMR, Kummer N, Di Fazio V, Ruyssinckx E, Samyn N (2013) Quantitative analysis of 26 opioids, cocaine, and their metabolites in human blood by ultra performance liquid chromatography–tandem mass spectrometry. Ther Drug Monit 35:510-521. https://doi.org/10.1097/FTD.0b013e31828e7e6b

Gerace E, Salomone A, Luciano C, Di Corcia D, Vincenti M (2018) First case in italy of fatal intoxication involving the new opioid U-47700. Front Pharmacol 9:747. https://doi.org/10.3389/fphar.2018.00747

Jamali B, Sheikholeslami B, Hosseinzadeh Ardakani Y, Lavasani H, Rouini MR (2017) Evaluation of the ecstasy influence on tramadol and its main metabolite plasma concentration in rats. Drug Metab Pers Ther 32:137–145. https://doi.org/doi:10.1515/dmpt-2017-0018

Kelly KR, Pypendop BH, Christe KL (2015) Pharmacokinetics of tramadol following intravenous and oral administration in male rhesus macaques (Macaca mulatta). J Vet Pharmacol Ther 38:375–382. https://doi.org/10.1111/jvp.12194

Knych HK, Corado CR, Mckemie DS, Steffey EP (2013) Pharmacokinetics and selected pharmacodynamic effects of tramadol following intravenous administration to the horse. Equine Vet J 45:490–496. https://doi.org/https://doi.org/10.1111/j.2042-3306.2012.00688.x

Matuszewski BK, Constanzer ML, Chavez-Eng CM (2003) Strategies for the assessment of matrix effect in quantitative bioanalytical methods based on HPLC-MS/MS. Anal Chem 75:3019-3030. https://doi.org/10.1021/ac020361s

McMillan CJ, Livingston A, Clark CR, Dowling PM, Taylor SM, Duke T, Terlinden R (2008) Pharmacokinetics of intravenous tramadol in dogs. Can J Vet Res 72:325–331

Meyer MR, Rosenborg S, Stenberg M, Beck O (2015) First report on the pharmacokinetics of tramadol and O-desmethyltramadol in exhaled breath compared to plasma and oral fluid after a single oral dose. Biochem Pharmacol 98:502–510. https://doi.org/https://doi.org/10.1016/j.bcp.2015.09.008

Nordmeier F, Doerr A, Laschke MW, et al (2020) Are pigs a suitable animal model for in vivo metabolism studies of new psychoactive substances? A comparison study using different in vitro/in vivo tools and U-47700 as model drug. Toxicol Lett 329:12-19. https://doi.org/https://doi.org/10.1016/j.toxlet.2020.04.001

Nordmeier F, Doerr AA, Potente S, et al (2021) Perimortem distribution of U-47700, tramadol and their main metabolites in pigs following intravenous administration. J Anal Toxicol (*submitted*)

Peters F, Paul L, Musshoff F, et al (2009) Anhang B zur Richtlinie der GTFCh zur Qualitätssicherung bei forensisch-toxikologischen Untersuchungen Anforderungen an die Validierung von Analysemethoden. Toxichem Krimtech. https://www.gtfch.org/cms/images/stories/files/GTFCh_Richtlinie_Anhang B_Validierung_Version 1.pdf. Accessed March 2021

Quetglas EG, Azanza JR, Cardenas E, Sádaba B, Campanero MA (2007) Stereoselective pharmacokinetic analysis of tramadol and its main phase I metabolites in healthy subjects after intravenous and oral administration of racemic tramadol. Biopharm Drug Dispos 28:19–33. https://doi.org/https://doi.org/10.1002/bdd.526

Schaefer N, Kettner M, Laschke MW, et al (2017) Distribution of synthetic cannabinoids JWH-210, RCS-4 and Δ 9-tetrahydrocannabinol after intravenous administration to pigs. Curr Neuropharmacol 15:713–723. https://doi.org/10.2174/1570159X15666161111114214

Schaefer N, Wojtyniak J-G, Kettner M, et al (2016) Pharmacokinetics of (synthetic) cannabinoids in pigs and their relevance for clinical and forensic toxicology. Toxicol Lett 253:7–16. https://doi.org/10.1016/J.TOXLET.2016.04.021

Schaefer N, Wojtyniak J-G, Kroell A-K, et al (2018) Can toxicokinetics of (synthetic) cannabinoids in pigs after pulmonary administration be upscaled to humans by allometric techniques? Biochem Pharmacol 155:403–418. https://doi.org/10.1016/J.BCP.2018.07.029

Seither J, Reidy L (2017) Confirmation of carfentanil, U-47700 and other synthetic opioids in a human performance case by LC-MS-MS. J Anal Toxicol 41:493-497. https://doi.org/10.1093/jat/bkx049

Smith CR, Truver MT, Swortwood MJ (2019) Quantification of U-47700 and its metabolites in plasma by LC-MS/MS. J Chromatogr B 1112:41–47. https://doi.org/https://doi.org/10.1016/j.jchromb.2019.02.026

United States Patent (2018). Intravenous administration of tramadol. US 10,022, 321 B2. Accessed March 2021.

Yılmaz B, Erdem AF (2015) Simultaneous determination of tramadol and its metabolite in human plasma by GC/MS. J AOAC Int 98:56–61. https://doi.org/10.5740/jaoacint.14-085
